# Quantified Morphology of the Cervical and Subdiaphragmatic Vagus Nerves of Human, Pig, and Rat

**DOI:** 10.3389/fnins.2020.601479

**Published:** 2020-11-04

**Authors:** Nicole A. Pelot, Gabriel B. Goldhagen, Jake E. Cariello, Eric D. Musselman, Kara A. Clissold, J. Ashley Ezzell, Warren M. Grill

**Affiliations:** ^1^Department of Biomedical Engineering, Duke University, Durham, NC, United States; ^2^Histology Research Core, The University of North Carolina at Chapel Hill, Chapel Hill, NC, United States; ^3^Department of Electrical and Computer Engineering, Duke University, Durham, NC, United States; ^4^Department of Neurobiology, Duke University, Durham, NC, United States; ^5^Department of Neurosurgery, School of Medicine, Duke University, Durham, NC, United States

**Keywords:** vagus nerve, vagus nerve stimulation, peripheral nerve stimulation, autonomic nerve stimulation, bioelectronic medicine, nerve morphology, perineurium, laboratory animal models

## Abstract

It is necessary to understand the morphology of the vagus nerve (VN) to design and deliver effective and selective vagus nerve stimulation (VNS) because nerve morphology influences fiber responses to electrical stimulation. Specifically, nerve diameter (and thus, electrode-fiber distance), fascicle diameter, fascicular organization, and perineurium thickness all significantly affect the responses of nerve fibers to electrical signals delivered through a cuff electrode. We quantified the morphology of cervical and subdiaphragmatic VNs in humans, pigs, and rats: effective nerve diameter, number of fascicles, effective fascicle diameters, proportions of endoneurial, perineurial, and epineurial tissues, and perineurium thickness. The human and pig VNs were comparable sizes (∼2 mm cervically; ∼1.6 mm subdiaphragmatically), while the rat nerves were ten times smaller. The pig nerves had ten times more fascicles—and the fascicles were smaller—than in human nerves (47 vs. 7 fascicles cervically; 38 vs. 5 fascicles subdiaphragmatically). Comparing the cervical to the subdiaphragmatic VNs, the nerves and fascicles were larger at the cervical level for all species and there were more fascicles for pigs. Human morphology generally exhibited greater variability across samples than pigs and rats. A prior study of human somatic nerves indicated that the ratio of perineurium thickness to fascicle diameter was approximately constant across fascicle diameters. However, our data found thicker human and pig VN perineurium than those prior data: the VNs had thicker perineurium for larger fascicles and thicker perineurium normalized by fascicle diameter for smaller fascicles. Understanding these differences in VN morphology between preclinical models and the clinical target, as well as the variability across individuals of a species, is essential for designing suitable cuff electrodes and stimulation parameters and for informing translation of preclinical results to clinical application to advance the therapeutic efficacy of VNS.

## Introduction

**FIGURE 1:**
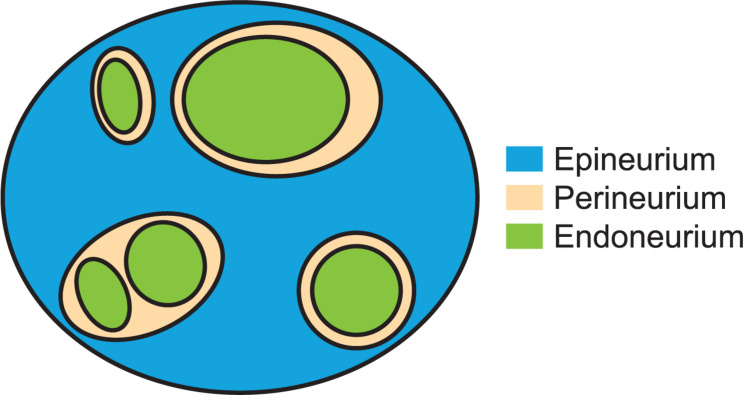
Cartoon of neural and connective tissues that were segmented and measured in this study. This example has three fascicles for which the perineurium thickness is a well-defined metric (with a single inner perineurium boundary for each outer boundary) and one “peanut” fascicle with multiple inner perineurium boundaries within a single outer boundary. The endoneurium includes all cells and tissues within the perineurial boundary.

The vagus nerve (VN) innervates most truncal organs and serves important roles in homeostatic regulation. The VN is also a surgically accessible point of intervention for electrical autonomic nerve stimulation to treat a range of diseases, including epilepsy (Ryvlin et al., 2014), depression (Müller et al., 2018), rheumatoid arthritis (Koopman et al., 2016), and obesity (Apovian et al., 2017). However, to design and deliver effective and selective vagus nerve stimulation (VNS), it is necessary to understand the morphology of the VN. Different neural and connective tissues (Figure 1) have different electrical conductivities (Geddes and Baker, 1967; Gabriel et al., 1996; Pelot et al., 2019), and the morphology of the nerve can influence patterns of stimulation. For example, thresholds for electrical activation or block with an implanted cuff electrode depend on the electrode-fiber distance, and thus on the nerve diameter; further, fascicle diameters, spatial arrangement of fascicles, and perineurium thickness all significantly influence thresholds (Koole et al., 1997; Grinberg et al., 2008; Pelot et al., 2019). Thus, data on nerve morphology are necessary to inform computational models to quantify these species- and nerve-specific responses to VNS (Helmers et al., 2012; Gómez-Tames et al., 2014; Arle et al., 2016; Mourdoukoutas et al., 2017; Pelot et al., 2017). In addition, morphological considerations are important in selecting appropriate animal models to evaluate and characterize neural stimulation therapies; such data will inform translation of stimulation parameters across species, including from preclinical studies to clinical applications.

There are published descriptions of vagal morphology, including from the human cervical VN (Seki et al., 2014; Hammer et al., 2015, 2018; Verlinden et al., 2016), human subdiaphragmatic VN (Tailai et al., 1980), rat cervical VN (Fazan and Lachat, 1997; Licursi de Alcântara et al., 2008), and rat subdiaphragmatic VN (Prechtl and Powley, 1985). However, each study focused on a single species, typically a single nerve level (e.g., cervical or subdiaphragmatic), and included only a subset of morphological metrics; further, there is a dearth of data on VN morphology in large animals. There are also data on the perineurium thickness in human somatic nerves (Sunderland and Bradley, 1952; Grinberg et al., 2008), although the method of identifying the perineurium in the original study is not clear, and the perineurium thicknesses in other species and in autonomic nerves are unknown.

We quantified the morphology of the cervical and subdiaphragmatic VN in humans, pigs, and rats. Healthy and disease model pigs are commonly used in preclinical studies, including for VNS (Tosato et al., 2007; Val-Laillet et al., 2010; Wolthuis et al., 2016; Nicolai et al., 2020), because their size and (patho)physiology approximate certain clinical settings, such as obesity and cardiac conditions, better than other animals (Johansen et al., 2001; Sauleau et al., 2009; Clouard et al., 2012; Swindle et al., 2012; Koopmans and Schuurman, 2015; Crisóstomo et al., 2016; Roura et al., 2016; Wolthuis et al., 2016; Bikou et al., 2018). Rats are commonly used as small animal models in neural stimulation studies (Borovikova et al., 2000; Waataja et al., 2011; Ward et al., 2015; Patel et al., 2017; McAllen et al., 2018; Morrison et al., 2018; Pelot and Grill, 2020). We quantified nerve size, number of fascicles, fascicle size, proportions of endoneurial, perineurial, and epineurial tissues, and perineurium thickness. The human and pig VNs were of comparable sizes, but the pig nerves had ten times more fascicles than human, while the rat nerves were ten times smaller in diameter. Human and pig VN perineurium was thicker than previously published human somatic nerve data; our data also showed thicker perineurium for larger fascicles, as well as thicker perineurium as a proportion of fascicle diameter for smaller fascicles. Species caused the greatest morphological differences, followed by individual variability.

## Materials and Methods

The data associated with this study are posted on the SPARC Portal (RRID:SCR_017041) at Pelot et al. (2020a, b, c, d, e, f, g).

### Sample Collections

We collected samples of cervical and subdiaphragmatic VNs from human cadavers, pigs, and rats. Specifically, we collected 37 cervical and 32 subdiaphragmatic human samples (*n* = 30 subjects, 15M/15F, 54 to 90+ years old, 63 ± 12 kg, 168 ± 10 cm in height); 21 cervical and 24 subdiaphragmatic pig samples (*n* = 12 pigs, 3M/9F, 10.5 to 15 weeks old, 33 ± 8 kg); and 18 cervical and 9 subdiaphragmatic rat samples (*n* = 10 rats, 5M/5F, 75 to 268 days old, 381 ± 118 g), where each subdiaphragmatic rat sample included the esophagus with both anterior and posterior VNs. Detailed metadata for each subject and each sample are provided in Supplement 1. The four nerve locations (right/left cervical and posterior/anterior subdiaphragmatic) were not sampled for all human subjects because the cadavers had already been dissected by medical trainees and thus were not always complete; full sample sets were collected in most cases for pigs and rats, with exceptions where the cervical nerve was not readily identified on one side in two pigs and where the subdiaphragmatic sample of one rat (R18) was highly unusual with multiple fascicles around a blood vessel that was not closely attached to the esophagus—unlike all other anterior trunks for all species—and thus another subdiaphragmatic sample (R26) was collected.

We collected VN samples from embalmed human cadavers. The study was deemed exempt by the Duke University Institutional Review Board. The bodies were donated to the Duke Anatomical Gifts Program, and we accessed them after they were used for medical training courses. The cadavers were embalmed with DUMC Embalming Fluid from the Carolina Biological Supply Company (33.3% ethanol, 13.2% phenol, 3.7% formaldehyde, 1% methyl isobutyl ketone, and 1% methanol). We collected fresh VN samples from adult Yorkshire (pink, domestic) pigs after they were euthanized following medical training courses or following other experiments approved by the Duke University Institutional Animal Care and Use Committee (IACUC). We collected VN samples from perfused adult Sprague-Dawley rats from Charles River that were used in other experiments approved by the Duke University IACUC; we performed transcardiac perfusions using at least 300 mL of PBS, followed by at least 300 mL of cold 4% paraformaldehyde.

For all species, we collected cervical VN samples bilaterally. In human cadavers, we collected 2 cm samples approximately where an imaginary line from the rostral end of the sternum to the earlobe intersected with the VN, also corresponding to the midlevel of the thyroid cartilage (laryngeal prominence). In pigs, we referenced an imaginary rostocaudal line from the level of the rostral end of the sternum to the level of the angle of the mandible (∼13 to 16 cm; see Supplementary Table 5); we collected 2 cm samples halfway along this line. In rats, we collected ∼6 to 8 mm samples where the VN courses straight with the common carotid artery, from the clavicle at the caudal end up to where the VN and carotid intermingle with other nerves. The sampling locations of the cervical VNs are consistent with the placement of VNS electrodes in clinical applications (Santos, 2004; Milby et al., 2008), preclinical studies in pigs (Tosato et al., 2007; Wolthuis et al., 2016; Nicolai et al., 2020), and preclinical studies in rats (Borovikova et al., 2000; Ward et al., 2015; Patel et al., 2017; McAllen et al., 2018; Morrison et al., 2018; Pelot and Grill, 2020). We excised rat cervical VNs attached to the common carotid artery (i.e., the entire carotid sheath) to provide additional mechanical stability during sample handling and embedding. We measured from the “valley” (i.e., apex) of the common carotid bifurcation to the center of each sample (mean ± SD: 30 ± 8 mm in humans; 62 ± 13 mm in pigs; 11 ± 1 mm in rats; see raw metadata in Supplementary Tables 4–6).

For all species, we collected samples of the anterior and posterior subdiaphragmatic VN trunks along the esophagus halfway between the diaphragmatic esophageal hiatus and the gastroesophageal junction, consistent with the placement of subdiaphragmatic VNS electrodes clinically (Camilleri et al., 2008; Ikramuddin et al., 2014) and in preclinical studies in pig (Wolthuis et al., 2016) and in rat (Waataja et al., 2011; Pelot and Grill, 2020). We sampled the subdiaphragmatic VNs in rats by collecting the full length of the subdiaphragmatic esophagus with the anterior and posterior vagal trunks attached to provide additional mechanical stability during sample handling and embedding. The human and pig samples were ∼2 cm long and the rat samples were 1.3 to 2.3 cm long.

### Histology

We stained all samples using Masson’s trichrome (MT) for initial visualization of the sample quality and morphology (Supplement 3). The MT histology provided sufficient contrast to segment the rat perineurium, as well as the pig endoneurium and epineurium. However, the human and pig perineurium required different approaches. Specifically, anti-claudin-1 immunohistochemistry (IHC) allowed more rapid and more objective segmentation of the human vagal perineurium than MT staining (Supplementary Figure 17), while anti-fibronectin immunofluorescence (IF) allowed segmentation of the pig vagal perineurium (Figure 4), which was otherwise challenging to identify in the MT micrographs, even for manual segmentation. The perineurium contains many known proteins, including claudin-1 and fibronectin (Jaakkola et al., 1989; Pummi et al., 2004; Peltonen et al., 2013; Zilic et al., 2015; Palladino et al., 2017; Reinhold and Rittner, 2017; Liu et al., 2018). However, we did not achieve strong and selective IHC reactivity with anti-claudin-1 in pigs; anti-claudin-1 in rats produced usable micrographs for segmentation, but there was background labeling and the segmentation process was comparable to analysis of the MT slides. Further, we did not achieve successful labeling with antibodies against other perineurial proteins when using IHC—GLUT-1 (tested in all species) or laminin (tested in pig)—despite evaluating different heat-induced epitope retrieval protocols and primary antibody concentrations (data not shown). The following paragraphs provide details of the histological methods.

After dissection, we sprayed each sample with mordant (ColorBond^TM^ Tissue Marking Dye Mordant, StatLab, McKinney, TX, United States), waited a couple of minutes, and dyed the rostral end of each sample green (Green Tissue Marking Dye, StatLab) to maintain orientation during processing. For the rat subdiaphragmatic samples, we also dyed the ventral surface of the esophagus green to distinguish the anterior and posterior nerves in the micrographs. We placed each sample between two histology sponges in a mega-sized histology cassette. We post-fixed each sample in 4% paraformaldehyde for 2 to 8 days (see Supplementary Tables 4–6) in a 4°C refrigerator. Following standard paraffin processing and embedding procedures, 5 μm sections were collected and placed on charged slides. After air drying overnight, slides were baked, deparaffinized, and hydrated to distilled water.

For the MT staining, we first placed the slides in Bouin’s fixative (mordant) at room temperature overnight. After rinsing the slides, we placed them in Weigert’s iron hematoxylin solution, Biebrich scarlet-acid fuchsin solution, and phosphomolybdic-phosphotungstic acid solution, with rinses in running tap water between each. We then placed them in aniline blue solution and differentiated the analine blue counterstain in 1% acetic acid solution. We dehydrated, cleared, and coverslipped the slides.

We conducted IHC to label selectively the perineurium using an antibody against the claudin-1 protein in the human nerve samples selected for morphological quantification. We conducted heat-induced epitope retrieval (HIER) at 120°C using a pH 6.0 buffer (TA-135-HBL, Thermo). We blocked endogenous peroxidases using 3% hydrogen peroxide followed by Dako Protein Block (X0909, Agilent). We incubated the slides in rabbit anti-claudin-1 (1:50, ab15098, Abcam, RRID:AB_301644) at 4°C overnight, followed by biotinylated SP-conjugated Affinipure goat anti-rabbit IgG (H+L) (1:500, #111-065-144, Jackson ImmunoResearch, RRID:AB_2337965) for 1 h at room temperature. We visualized staining using a Vectastain Elite ABC HRP kit (Vector Laboratories, PK-6100) followed by DAB chromogen (TA-125-QHDX, Thermo). Finally, we counterstained the slides with hematoxylin (6765003, Thermo Fisher) and coverslipped using DPX (13512, EMS). We prepared control samples (one cervical and one subdiaphragmatic) by eliminating the primary antibody. The claudin-1 antibody was raised against a synthetic peptide within human claudin-1 aa 150 to the C-terminus. The ab15098 antibody was used for targeting peripheral nerve perineurium (Cohnen et al., 2020), as well as for applications of IHC of many other paraffin-embedded tissues [e.g., skin (Kim et al., 2016), intestine (Gumber et al., 2014; Mandle et al., 2019), and kidney (Koda et al., 2014)]; other claudin-1 antibodies were also used to label perineurium in peripheral nerves (Pummi et al., 2004; Rittner et al., 2012; Piña et al., 2015; Reinhold et al., 2018).

We conducted IF on the pig nerve samples selected for morphological quantification to label selectively the perineurium using an antibody against the fibronectin protein. We permeabilized the sections with 0.1% Triton X-100 (v/v, BP151-500, Fisher Scientific) in PBS for 20 min, followed by incubation in 7.5% bovine serum albumin (w/v, BSA, Sigma) in PBS for 1 h. We then incubated the slides in rabbit anti-fibronectin (1:50, F3648, Sigma, RRID:AB_476976) at 4°C overnight, followed by goat polyclonal secondary antibody to rabbit IgG (H+L, FITC) (1:100, ab97050, Abcam, RRID:AB_10698224) for 1 h at room temperature. We coverslipped the slides with Fluoro-Gel II with DAPI (17985-50, EMS). We prepared two slides for each control sample (one cervical and one subdiaphragmatic): one slide excluded the primary antibody and the other excluded both the primary and secondary antibodies. The fibronectin antibody was raised against fibronectin isolated from human plasma and was used to label perineurium in pig sciatic nerve (Zilic et al., 2015).

### Imaging and Image Analysis

We used a Nikon Ti2 inverted microscope for all imaging (Nikon Instruments Inc., Tokyo, Japan). We imaged the pig MT, human MT, and human anti-claudin-1 slides at 10× (Plan Apochromat Lambda, NA: 0.45) and the rat MT slides at 20× (Plan Apochromat Lambda, NA: 0.75), all using a DS-Ri2 color CMOS camera (Nikon Instruments Inc.). We imaged the pig anti-fibronectin slides at 20× with a GFP/FITC/cy2 filter set (excitation: 466/40 nm (446–486 nm), emission: 525/50 nm (500–550 nm), dichroic mirror: 495 nm; Nikon Instruments Inc.), a SOLA SE II 365 light engine (Lumencor, Beaverton, OR, United States), and a Photometrics Prime 95B-25MM camera (Teledyne Photometrics, Tucson, AZ, United States). For each sample, we selected the best of the stained sections based on the lack of tearing or fraying.

We selected a subset of our VN micrographs for image analysis. We quantified the morphology of nine left cervical and nine anterior subdiaphragmatic samples for each species, using claudin-1 IHC for the human samples and MT staining for the pig and rat samples. We also quantified the morphology for four cervical (2F/2M) and four subdiaphragmatic (2F/2M) pig samples using anti-fibronectin IF; these eight images were selected from the 18 pig samples for their clearer contrast between the perineurium and surrounding tissues (Supplementary Figure 15). After excluding human subjects with known neuropathies (Supplementary Table 1), within each sex, we randomly selected left cervical (4F/5M) and anterior subdiaphragmatic (4F/5M) human samples for further analysis. We analyzed all left cervical VN samples from pigs (6F/3M), as well as all anterior subdiaphragmatic samples from male pigs (3M); we randomly chose six of the nine anterior subdiaphragmatic samples from female pigs (6F). We analyzed all left cervical (4F/5M) and anterior subdiaphragmatic (4F/5M) rat VN samples.

We segmented the endoneurial, perineurial, and epineurial tissues (Figure 1) as applicable for each micrograph using Nikon’s NIS-Elements Ar software (v5.02.01, Build 1270, Nikon Instruments Inc.). For the rat MT micrographs (Figure 5), we used the manual segmentation tools in NIS-Elements. For the rat cervical VN samples, we identified the vagus as the largest nerve in the carotid sheath (also see Discussion). For the rat subdiaphragmatic VN samples, we identified and segmented the largest fascicle on the anterior surface of the esophagus; we also segmented smaller fascicles nearby if they were within a distance of approximately twice the diameter of the largest fascicle from the edge of the largest fascicle, as well as an associated nerve boundary where denser connective tissue transitioned to looser connective tissue, to approximate the tissues that would be within a cuff electrode. However, for all statistics, we only used the largest fascicle in each rat sample, using the outer perineurial boundary as the nerve boundary, to allow consistent comparisons between samples since the rat subdiaphragmatic fascicles were not connected by distinct collagenous epineurium like the pig and human samples.

**FIGURE 2:**
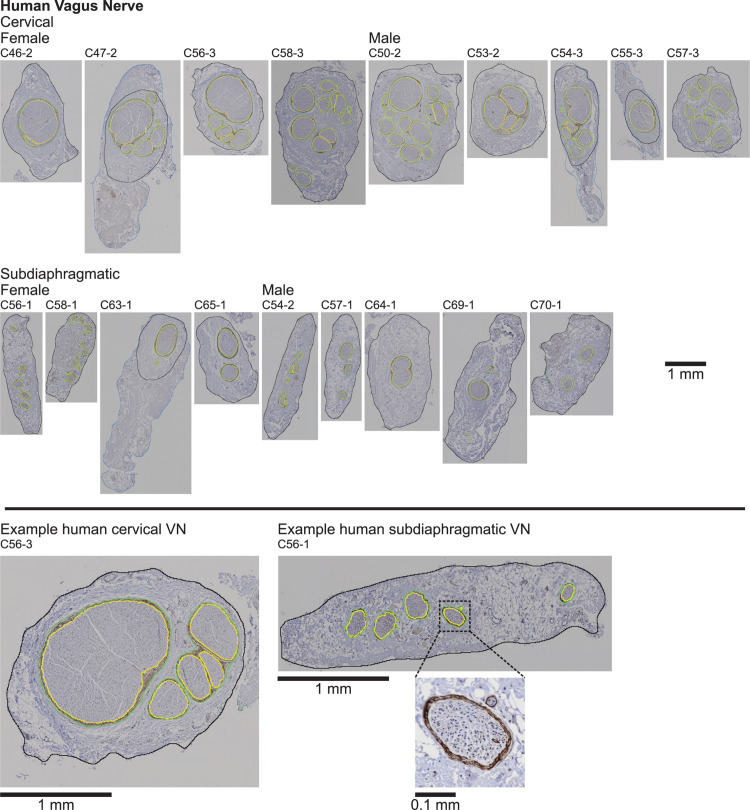
Human vagus nerve samples with claudin-1 immunohistochemistry labeling and overlaid segmentation of the nerve and perineurium. Note in four samples (C47-2, C54-3, C55-3, and C63-1), the outer nerve segmentation is shown capturing all of the tissue in the micrograph (blue), but we quantified the morphology using the inner nerve trace (black), excluding extraneous tissue. In some samples, we observed what we dubbed “peanut fascicles,” where a single outer perineurium trace (green) contained multiple fiber bundles separated by an interior septum, resulting in multiple inner perineurium traces (yellow), e.g., the right-hand green trace containing three yellow traces in the enlarged example cervical sample (C56-3; bottom left). No immunoreactivity was observed in the no primary controls (Supplementary Figure 13). All original micrographs and segmentations are available at Pelot et al. (2020a).

**FIGURE 3:**
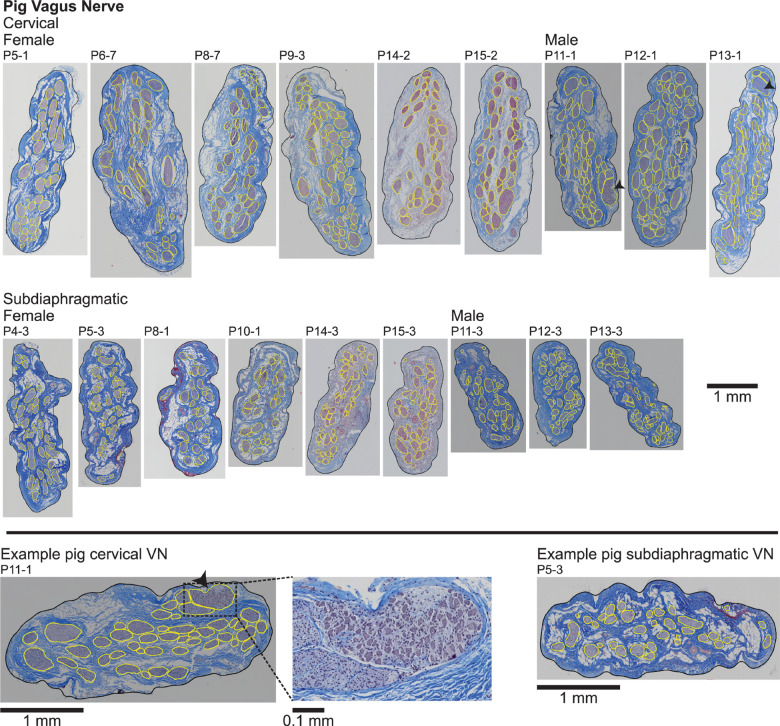
Pig vagus nerve samples with Masson’s trichrome staining and overlaid segmentation of the nerve and endoneurium (inner perineurium boundaries). The black arrowheads mark fascicles containing cell bodies in two cervical samples (P11-1 and P13-1), as shown in the zoomed view for P11-1 (bottom middle). All original micrographs and segmentations are available at Pelot et al. (2020d, f).

**FIGURE 4:**
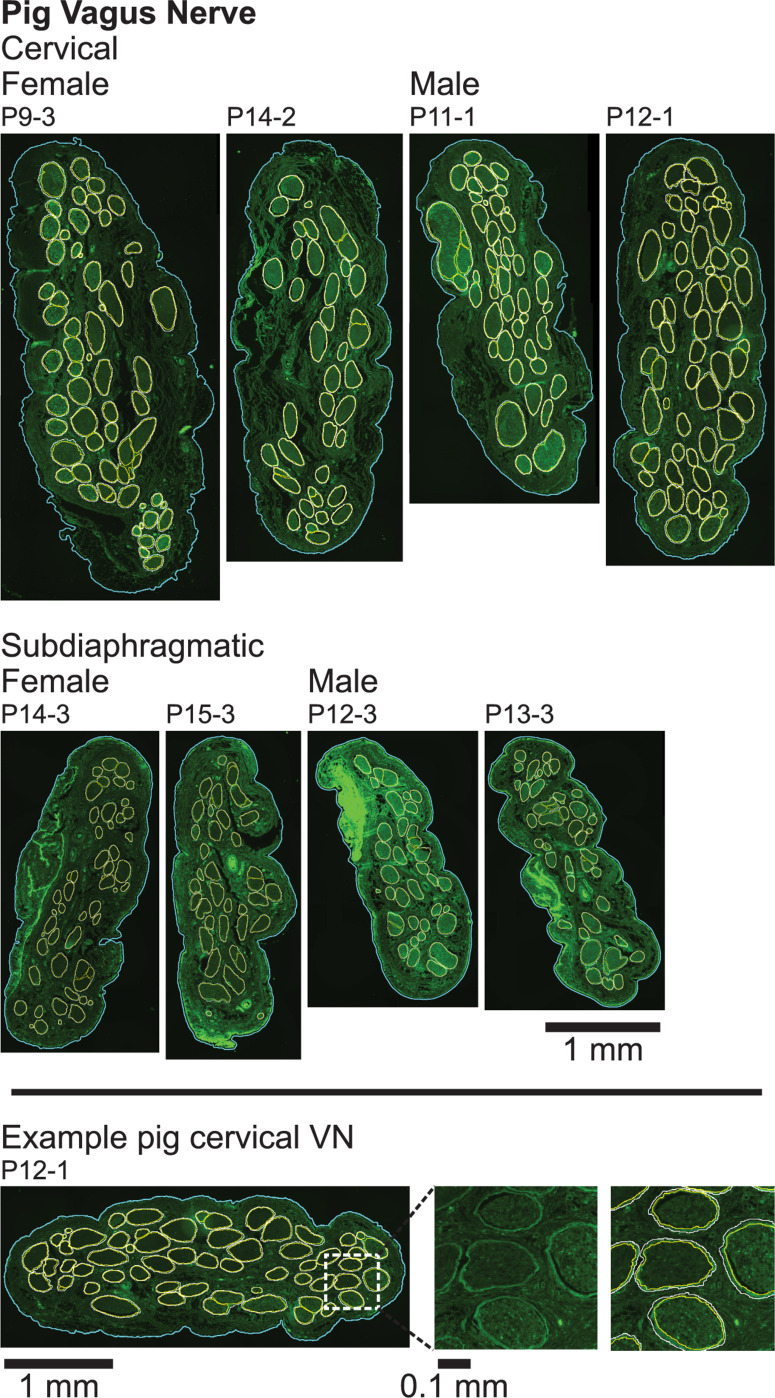
Pig vagus nerve samples with anti-fibronectin immunofluorescence and overlaid segmentation of the perineurium and outer nerve boundary. No perineurial immunoreactivity above background levels was observed in the pig no primary and no primary/no secondary controls (Supplementary Figure 15). All original micrographs and segmentations are available at Pelot et al. (2020b).

For the human claudin-1 IHC, pig MT, and pig anti-fibronectin IF micrographs (Figures 2–4), we used the General Analysis tool in NIS-Elements. For each image, we performed pre-processing (smoothing, sharpening), segmentation (setting of appropriates bounds for the hue, saturation, intensity), and post-processing (minimum size criterion, closing, smoothing, dilating, and eroding). We defined the settings of this image analysis protocol for each species (humans and pigs), each level (cervical and subdiaphragmatic), and each target tissue (whole nerve, as well as perineurial ring for the human IHC and pig IF, but inner perineurium boundary for the pig MT micrographs; see details below on the latter). We then manually edited each segmented image to fill holes and to remove spurs and off-target elements such as blood vessels.

We manually segmented the perineurial ring for one cervical and one subdiaphragmatic pig MT micrograph. For the main set of 18 pig MT micrographs, an additional step was required for the analysis of the fascicles (Figure 3) because the perineurium was very thin and not readily identified; therefore, instead of segmenting the perineurial ring, we instead segmented the endoneurium (i.e., inner perineurial boundary). Using the above General Analysis protocol, the pig endoneurium was well-defined. However, due to shrinkage during sample processing, the endoneurium was sometimes detached from the inner perineurium boundary. To address this issue and capture both the endoneurium and the immediately adjacent whitespace, we created a secondary image processing layer that identified the image whitespace using intensity-based segmentation. We slightly inflated this segmented whitespace and identified objects in the whitespace layer that intersected with the endoneurial layer. We merged these identified objects with the original endoneurial layer and manually edited the binary objects through cutting and filling operations. This resulted in a segmented layer identifying the inner perineurial boundaries (Supplement 2).

We exported the resulting binary segmented images from NIS-Elements as TIFs (“graticule masks”) and imported them into MATLAB R2018b (MathWorks, Inc., Natick, MA, United States). We identified boundaries using the *bwboundaries* function to quantify morphology. We calculated the surface area within each boundary using the *polyarea* function and calculated the effective diameter (for the nerve and fascicles) as the diameter of a circle with the same area. We estimated the perineurium thickness as half of the difference between the effective diameters of the inner and outer perineurium boundaries. We quantified the effective fascicle diameters using the inner perineurium boundaries.

### Statistics and Data Analyses

All variability measures are provided as standard deviations (SDs). We performed mixed model ANOVAs in JMP^®^ Pro 14.3.0 (SAS Institute Inc., Cary, NC, United States). For effective nerve diameter and number of fascicles, we included fixed effects of species (human, pig, and rat for nerve diameter; only human and pig for number of fascicles) and nerve level (cervical, subdiaphragmatic), as well as the random effect of subject number; we excluded rats from the analysis of number of fascicles because we only considered the largest fascicle in each sample. For fascicle diameters, we took the common logarithm of the data, and we also included the random effect of sample number, since there were multiple fascicle measurements per nerve in the cases of multifascicular samples. We followed the ANOVAs with Tukey’s HSD test for *post hoc* analyses. We fit the perineurium thickness data in MATLAB using the *fit* function with the linear least squares method.

## Results

**FIGURE 5:**
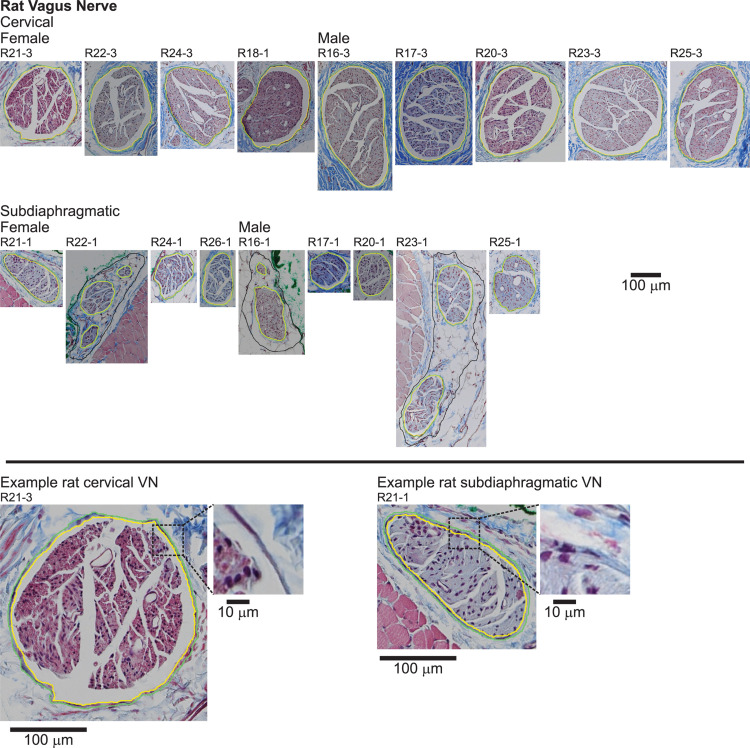
Rat vagus nerve samples with Masson’s trichrome staining and overlaid segmentation of the nerve and perineurium. All original micrographs and segmentations are available at Pelot et al. (2020e, g).

Cross sections of all collected VN samples stained with MT are shown in Supplement 3, and detailed metadata for each subject and each sample are provided in Supplement 1. We segmented the gross morphology (nerve, outer perineurium, and inner perineurium boundaries) of nine cervical and nine subdiaphragmatic VN samples from humans, pigs, and rats. These data are important for the design and translation of neuromodulation therapies because thresholds for activation and block vary with electrode-fiber distance (and thus, nerve diameter), fascicle diameter, spatial arrangement of fascicles, and perineurium thickness. For the human samples, we segmented anti-claudin-1 IHC micrographs (Figure 2), which provided much clearer distinction of the perineurial rings as compared to the MT staining. For the pig samples, we segmented the nerve and inner perineurium boundaries of the MT micrographs (Figure 3); in two of the pig cervical cross sections, one fascicle contained cell bodies, although we did not sample the nerve close to the nodose ganglion (Settell et al., 2020). We also segmented four cervical and four subdiaphragmatic anti-fibronectin pig IF micrographs which allowed distinction of the inner and outer perineurium boundaries (Figure 4), unlike the MT staining. Finally, for the rat samples, we segmented the MT micrographs (Figure 5).

**FIGURE 6:**
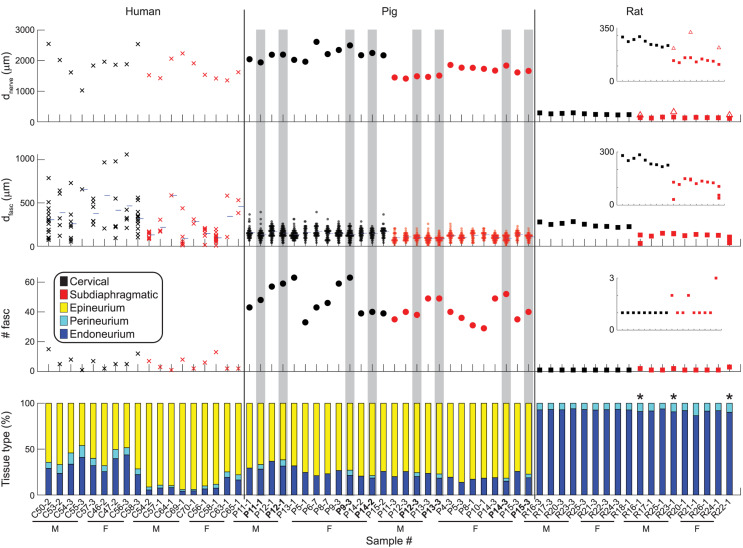
Quantitative morphology of individual vagus nerve samples from humans (left), pigs (middle), and rats (right). Eight of the pig samples are duplicated as they underwent both MT segmentation (as used for all pig samples, where the perineurium was not segmented) and anti-fibronectin IF segmentation (highlighted in gray, with bolded labels). Insets are provided for the rat data with more constrained *y*-axes. First row: Effective nerve diameter, i.e., diameter of a circle with the same cross-sectional area as the original nerve trace. For the three multifascicular rat subdiaphragmatic VNs, the red square is the effective diameter of the largest fascicle (outer perineurium boundary); the open triangle is the effective diameter of the connective tissue identified surrounding the fascicles. Second row: Effective fascicle diameters, i.e., diameter of circles with the same cross-sectional areas as the original inner perineurial traces. These diameters include those for fiber bundles within fascicles with more than one inner perineurium trace for a given outer perineurium trace (peanut fascicles); thus, these diameters include all inner perineurial traces. The blue horizontal ticks mark the mean fascicle diameter for each sample. Third row: Number of fascicles. These numbers include fiber bundles within peanut fascicles, i.e., total number of inner perineurial traces. Fourth row: Proportion of nerve cross-sectional area that is endoneurium, perineurium, and epineurium. The three rat samples with asterisks are multifascicular; the plotted proportions are for the largest fascicle to allow clearer comparison across the samples. The “Imaging and Image Analysis” section of the Materials and Methods details our approach for identifying rat subdiaphragmatic fascicles. If the multiple fascicles and surrounding tissue were included, the proportions of epineurium, perineurium, and endoneurium would be: 58.0, 3.8, and 38.2% for R16-1; 55.1, 4.2, and 40.7% for R23-1; 63.4, 3.6, 33.0% for R22-1. All original data are available at Pelot et al. (2020a, f, g, b).

**FIGURE 7:**
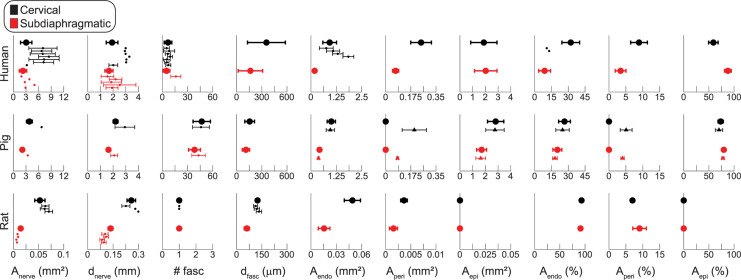
Summary statistics of vagus nerve morphology for human (top row), pig (middle row), and rat (bottom row) samples. The cervical data are in black and the subdiaphragmatic data are in red. The *x*-axes for human and pig data are matched; the rat axes are different to accommodate the smaller samples. The larger dots and thicker error bars are the data from our samples (mean ± SD), while the smaller dots, with thin error bars as available, are data from literature (Supplement 4); the triangles with thin error bars in the pig row are the anti-fibronectin IF data, while the large dots in the pig row are the MT data. The number of fascicles (# fasc) for human micrographs and for pig IF micrographs include fiber bundles with more than one inner perineurium trace for a given outer perineurium trace (peanut fascicles); thus, the number of fascicles counts the total number of inner perineurial traces. The rat data only account for the largest fascicle in each sample; see Figure 6 for multifascicular data. *A*_nerve_: total cross-sectional area of the nerve; *d*_nerve_: effective circular diameter of the nerve; # fasc: number of fascicles; *d*_fasc_: effective circular diameter of each fascicle; *A*_endo_: cross-sectional area covered by the endoneurium, i.e., area within the inner perineurial traces; *A*_peri_: cross-sectional area covered by the perineurium; *A*_epi_: cross-sectional area covered by the epineurium, where *A*_epi_ = *A*_nerve_ – *A*_endo_ – *A*_peri_. From top to bottom, the human cervical data from literature for *A*_nerve_ and *d*_nerve_ are from Hammer et al. (2015) (left VN), (Hammer et al., 2015) (right VN), (Verlinden et al., 2016) (left VN), (Verlinden et al., 2016) (right VN), (Hammer et al., 2018) (left VN), (Hammer et al., 2018) (right VN), (Stakenborg et al., 2020) (right and left VN), and the subdiaphragmatic data are from Tailai et al. (1980) (anterior VN), (Tailai et al., 1980) (posterior VN), (Gravgaard, 1968) (anterior VN), (Gravgaard, 1968) (posterior VN), (Stakenborg et al., 2020) (posterior VN). The human # fasc data are from Verlinden et al. (2016) (left VN), (Verlinden et al., 2016) (right VN), (Seki et al., 2014) (left VN), (Seki et al., 2014) (right VN), (Hammer et al., 2018) (left VN), (Hammer et al., 2018) (right VN), (Stakenborg et al., 2020) (right and left VN) at the cervical level, and (Stakenborg et al., 2020) (posterior VN) at the subdiaphragmatic level. The human *A*_endo_ data are from the same first four references as the # fasc data where plotted in mm^2^ and from the same first two references where plotted as a percentage. All pig data from literature were from Stakenborg et al. (2020). For the rat data, top to bottom, the cervical data for *A*_nerve_ and *d*_nerve_ are from Fazan and Lachat (1997) (right VN), (Licursi de Alcântara et al., 2008) (left VN), (Licursi de Alcântara et al., 2008) (right VN), and the subdiaphragmatic data are all from Prechtl and Powley (1985) (anterior VN at diaphragm, posterior VN at diaphragm, anterior gastric branch, posterior gastric branch), where they analyzed individual fascicles. The rat # fasc data are from the same first two references and the *d*_fasc_ data are from the same first three references.

Figure 6 shows metrics of nerve morphology for each sample: effective nerve diameter, effective fascicle diameters, number of fascicles, and proportions of endoneurium, perineurium, and epineurium. Effective diameter indicates the diameter of the circle with the same surface area as the original trace. Summary statistics for those metrics, as well as for cross-sectional area in mm^2^ for each tissue type, are shown in Figure 7, with comparisons to published data. A spreadsheet with our summary statistics for each species and nerve level, as well as summary statistics from literature, is provided in Supplement 4. The pig samples that underwent both MT and anti-fibronectin IF segmentation had comparable metrics (Figure 6).

The nerve diameter differed across species (*p* < 0.0001; *F* = 577.1) and nerve levels (*p* = 0.0030; *F* = 11.01), but we did not detect an interaction of species and level (*p* = 0.1085; *F* = 2.445). The human and pig nerves had similar effective diameters (1.9 ± 0.5 vs. 2.2 ± 0.2 mm at the cervical level and 1.7 ± 0.3 vs. 1.6 ± 0.2 mm at the subdiaphragmatic level; *p* = 0.1830, Tukey’s test), while the rat nerves were approximately ten times smaller (0.260 ± 0.025 mm at the cervical level and 0.135 ± 0.015 mm at the subdiaphragmatic level; *p* < 0.0001, for both comparisons of rat to larger species). The cervical nerves were significantly larger than the subdiaphragmatic nerves; qualitatively, this trend was clearest for the pig and rat nerves.

The number of fascicles differed across species (*p* < 0.0001; *F* = 213.8) and nerve levels (*p* = 0.0300; *F* = 5.486), but we did not detect an interaction of species and level (*p* = 0.1322; *F* = 2.481); we excluded the rat data because only the largest fascicle of each sample was included in the statistical analyses. The pig nerves had almost ten times more fascicles than the human nerves (47 ± 10 at the cervical level and 38 ± 7 at the subdiaphragmatic level for pigs vs. 6.7 ± 4.5 and 4.9 ± 4.0 for humans), and there were more fascicles at the cervical level.

The fascicle diameters differed across species (*p* = 0.0015; *F* = 8.989) and nerve levels (*p* < 0.0001; *F* = 51.88), and there was an interaction of species and level (*p* = 0.0121; *F* = 5.273). Comparing across species at the cervical level, the human fascicles (0.355 ± 0.226 mm) were larger than the pig fascicles (0.155 ± 0.058 mm; *p* = 0.0002), while rat fascicles (0.249 ± 0.024 mm) were comparable sizes to both human (*p* = 0.7270) and pig (*p* = 0.1707) fascicles. At the subdiaphragmatic level, the fascicles diameters were comparable across all species (human: 0.163 ± 0.146 mm; pig: 0.110 ± 0.044 mm; rat: 0.121 ± 0.032 mm; *p* = 0.3049 for human vs. pig; *p* = 0.8960 for human vs. rat; *p* = 0.9947 for pig vs. rat), although the human fascicles trended larger than the pig fascicles. All species had larger fascicles at the cervical level (*p* < 0.0001 for humans; *p* = 0.0009 for pigs; *p* = 0.0234 for rats).

Overall, while the pig approximated the human nerve in diameter, it had more fascicles, and the fascicles were smaller, particularly at the cervical level, as reflected by the similar proportion of endoneurium at the cervical level between pigs and humans, but smaller proportion for humans at the subdiaphragmatic level (Figure 6). The monofascicular rat nerves were ∼10× smaller, and their diameters approximated the diameter of a single human or pig fascicle.

Qualitatively, the nerve and fascicle diameters were more consistent across samples for the pig nerves than for the humans nerves (Figure 6), as expected given the variability in age, weight, height, lifestyle, health status, etc. across the human subjects. Further, pig nerves had consistent collagenous epineurium, whereas many human subdiaphragmatic nerves had fatty extrafascicular tissue (Supplement 3), and pig nerves were consistently oblong in shape, whereas the human samples were more circular at the cervical level (Figures 2, 3, Supplement 3). The human endoneurium occupied a larger proportion of the nerve at the cervical level (32 ± 7.9%) than at the subdiaphragmatic level (8.8 ± 5.3%) whereas the endoneurial proportions were more consistent across levels in pigs, slightly lower than in the human cervical VN (27 ± 5.3% at the cervical level and 20 ± 4.0% at the subdiaphragmatic level for the MT micrographs; means were ∼2 percentile points lower for the anti-fibronectin IF micrographs for which the segmentation accounted for the perineurium). These same trends between species and levels were present for the proportion of perineurium (8.9 ± 2.5% and 3.4 ± 1.6% at the cervical and subdiaphragmatic levels in humans; 5.1 ± 1.8% and 4.0 ± 0.5% in pigs). Finally, for the morphological data on rat subdiaphragmatic samples in Figure 6, we focused on the largest fascicle of each sample to allow a consistent comparison across samples given that there was not typical collagenous epineurium defining clear multifascicular nerves (Figure 5 and Supplement 3) (Pelot et al., 2020e); however, as detailed in the Materials and Methods, we also segmented other fascicles in the rat subdiaphragmatic micrographs if they were within a distance of approximately twice the diameter of the largest fascicle to approximate the neural tissue that could be captured within a cuff electrode.

We compared our morphological measurements to those reported previously in the literature (Figure 7 and Supplement 4), including data published for both left/right and anterior/posterior VNs, as specified in the caption. The numbers of fascicles and endoneurial cross-sectional areas of the human cervical VNs were comparable to previous reports, but the nerve cross-sectional areas and effective diameters were generally smaller, albeit with overlapping ranges. Consequently, we estimated a larger proportion of endoneurium over the cross section. The cross-sectional areas and effective diameters of human subdiaphragmatic VNs compared well to literature, particularly comparing to the published measurements for the anterior VN; we counted fewer fascicles than one prior study that examined the posterior branch. Our numbers of fascicles for the pig VN were consistent with literature, although the published data were for the right cervical and posterior subdiaphragmatic VNs; our mean nerve diameters were slightly smaller than the published means. The nerve diameters and cross-sectional areas of the rat cervical VNs compared well to published data, while our rat subdiaphragmatic nerves were slightly larger than prior data; we compared to data for the rat VN both at the level of the diaphragm and at the gastric branch, given that subdiaphragmatic branching patterns vary between animals (Prechtl and Powley, 1985).

**FIGURE 8:**
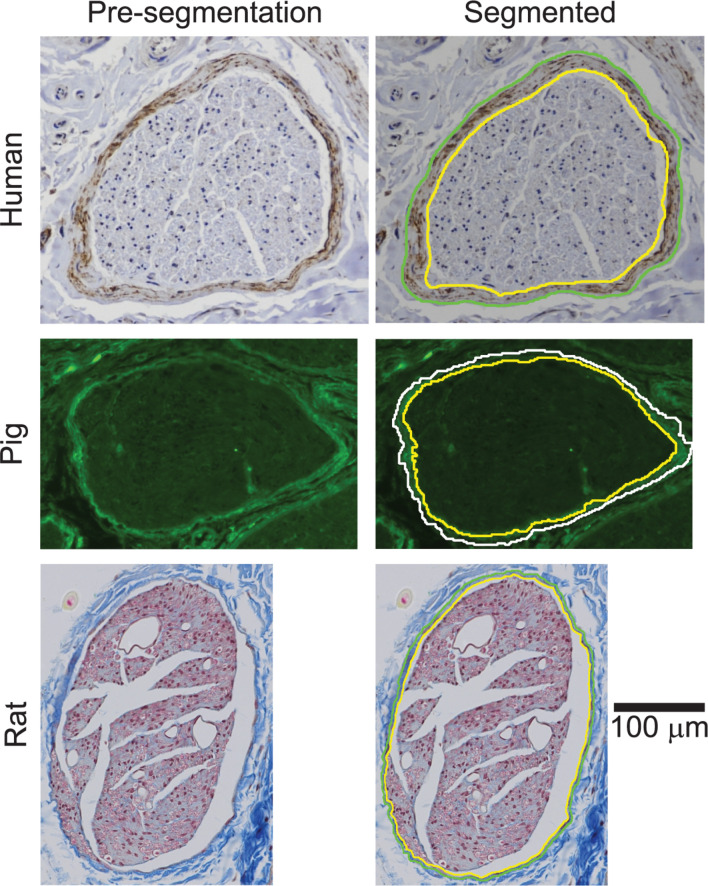
Example vagus nerve fascicles from human (anti-claudin-1 IHC; C57-3), pig (anti-fibronectin IF; P12-1), and rat (MT; R25-3), all approximately 250 μm in diameter. The left-hand column shows the raw image, with the contrast of the perineurium labeling relative to surrounding tissues; the right-hand column shows the resulting segmented traces to identify the inner and outer perineurium boundaries, where the human perineurium is the thickest and the rat perineurium is the thinnest.

Perineurium is a thin sheath of connective tissue, and due to its high resistivity relative to surrounding tissues (Weerasuriya et al., 1984; Pelot et al., 2019), its thickness has an important effect on the activation thresholds of its enclosing fibers in response to extraneural electrical signals (Grinberg et al., 2008). We quantified the perineurium thickness of fascicles in the VN for the three species, using the anti-claudin-1 IHC for human samples, anti-fibronectin IF for pig samples, and MT for rat samples (Figure 8).

**FIGURE 9:**
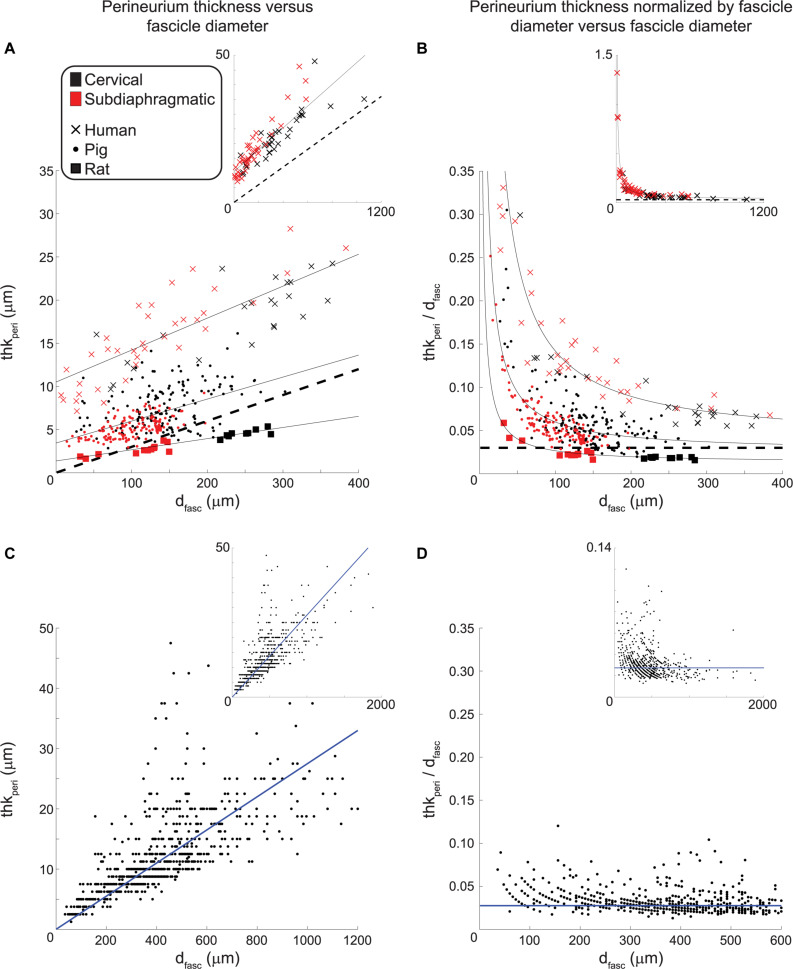
Perineurium thickness **(left)** and perineurium thickness normalized by fascicle diameter **(right)** plotted as functions of effective fascicle diameter for our VN data **(top)** and the somatic nerve data in Sunderland and Bradley (1952) **(bottom)**. The black dashed lines in the top row indicate a perineurium thickness that is 3% of the fascicle diameter, as found in Grinberg et al. (2008) by analyzing the data from Sunderland and Bradley (1952). The solid black lines indicate the fits for each species (Eqs. 1–3). The human and pig data exclude cases where there is more than one inner perineurium boundary for a given outer perineurium boundary (i.e., peanut fascicles) as the perineurium thickness is not a well-defined metric in those cases. For the human anti-claudin-1 IHC micrographs, the cervical level had 60 fascicles total (inner perineurium traces) of which 27 were intrafascicular bundles in 10 peanut fascicles; the subdiaphragmatic level had 44 fascicles total (inner perineurium traces) of which 4 were intrafascicular bundles in 2 peanut fascicles. For the pig anti-fibronectin IF micrographs, the cervical level had 210 fascicles total (inner perineurium traces) of which 57 were intrafascicular bundles in 25 peanut fascicles; the subdiaphragmatic level had 179 fascicles total (inner perineurium traces) of which 48 were intrafascicular bundles in 19 peanut fascicles. We fit the data from Sunderland and Bradley (1952) with a linear equation with forced zero intercept (Eq. 4). Note that the main plots in panels (A) and (B) do not show all human data (see insets), and the inset in panel (C) excludes two outliers at fascicle diameters of 630 and 1590 μm with corresponding perineurium thicknesses of 62.5 and 100 μm. All original data are available at Pelot et al. (2020a, g, b).

We plotted the perineurium thickness and the perineurium thickness normalized by fascicle diameter versus the effective fascicle diameters (Figures 9A,B, respectively). By fitting these data, the resulting equations can be used to define perineurium thickness in computational models of autonomic nerves of these species. For a given fascicle diameter, the human samples had the thickest perineurium while the rat samples had the thinnest perineurium. The ratio of perineurium thickness to fascicle diameter decreased with increased fascicle diameter, particularly for the human and pig samples (Figure 9B). We fit the data from 73 human fascicles (excluding fascicles with more than one inner perineurium trace for a given outer perineurium trace, i.e., peanut fascicles) as:

(1) $$thk_{\text{peri}}=0.03702\text{*}d_{\text{fasc}}+10.50$$

with *d*_fasc_ in microns, yielding *R*^2^ = 0.75. We fit the data from 284 pig non-peanut fascicles as:

(2) $$thk_{\text{peri}}=0.02547\text{*}d_{\text{fasc}}+3.440$$

with *d*_fasc_ in microns, yielding *R*^2^ = 0.30. Note that the fascicle diameters for the pig anti-fibronectin IF (Figures 9A,B; 14 to 303 μm, mean = 123 μm, median = 123 μm, interquartile range = 91 to 155 μm) are consistent with the MT data (Figure 6; 5 to 399 μm, mean = 135 μm, median = 130 μm, interquartile range = 96 to 168 μm). Further, the pig perineurium segmented from the anti-fibronectin micrographs is consistent with the data from two MT micrographs (one cervical and one subdiaphragmatic) that were manually segmented (Supplementary Figure 18). We fit the data from 22 rat fascicles as:

(3) $$thk_{\text{peri}}=0.01292\text{*}d_{\text{fasc}}+1.367$$

with *d*_fasc_ in microns, yielding *R*^2^ = 0.77. The data did not suggest a difference in the relationship between perineurium thickness and fascicle diameter for cervical versus subdiaphragmatic nerves for any of the three species. We also fit the data from Sunderland and Bradley (1952) for human somatic nerves with a linear equation with forced zero intercept:

(4) $$thk_{\text{peri}}=0.0275\text{*}d_{\text{fasc}}$$

with *d*_fasc_ in microns, yielding *R*^2^ = 0.51, to recreate the data analyses conducted in Grinberg et al. (2008).

## Discussion

VNS is used clinically to treat a broad spectrum of diseases and is investigated in preclinical models to determine mechanisms of action, to improve current therapies, and to evaluate new applications. Morphological differences in nerve diameter, fascicle diameter, number of fascicles, spatial arrangement of fascicles, and perineurium thickness all significantly affect activation thresholds and other biophysical responses of nerve fibers to electrical signals delivered by a cuff electrode. Larger diameter nerves will contain fibers that are further from the cuff electrode, and all else being equal (such as fiber diameter and fascicular structure), fibers will have higher thresholds for larger electrode-fiber distances (Jankowska and Smith, 1973; Follett and Mann, 1986; Pelot et al., 2017). Fibers within larger fascicles have higher thresholds (Grinberg et al., 2008; Pelot et al., 2017), and thicker (or more resistive) perineurium results in higher thresholds (Grinberg et al., 2008; Pelot et al., 2019). Further, the size and spatial arrangement of surrounding fascicles affect thresholds; for example, in the case of a nerve with two fascicles, the thresholds for fibers in fascicle A will be lower if fascicle B is larger (Grinberg et al., 2008). Thus, understanding differences in VN morphology between individuals of a given species and between preclinical models and the clinical target are paramount to advancing therapeutic efficacy. These quantitative morphological data will also inform computational models of VNS to allow systematic quantification of intra- and inter-species differences in response to stimulation.

With respect to within-species effects (Figure 6), the variability in the nerve diameters across human samples and in the fascicle diameters within and across human samples may contribute to differences in therapeutic parameter settings between patients (Heck et al., 2002). Although the number of fascicles varied between pig nerves, the nerve diameters and ranges of fascicle diameters were more consistent between samples for pig and rat nerves than for humans. We sampled the cervical and subdiaphragmatic VNs at standardized locations; therefore, morphological differences between individuals may partially reflect differences in VN branching patterns. Specifically, different branching patterns may be associated with different fascicular organizations, given that at the branching point, certain fascicles must separate from the main trunk. Further, different branching patterns may have affected the collected samples in some cases; for instance, in cadavers, when we observed multiple subdiaphragmatic trunks, we focused on the largest trunk since it would be the target for an implanted electrode. At the cervical level, we recorded the locations of the samples with respect to the bifurcation of the common carotid artery (Supplementary Tables 4–6), although there are variations of the level of bifurcation with respect to vertebral levels (Lo et al., 2006; Standring and Gray, 2009, p. 444). The human cervical VN can occasionally branch in the cervical region, with branches in 12 and 22% of cases on the left and right sides, respectively, innervating the “inferior larynx” and the “upper mediastinum” (Hammer et al., 2015). It is unclear whether these branches are unusual locations for common vagal branches (e.g., superior laryngeal, recurrent laryngeal) or additional vagal branches. At the subdiaphragmatic level, we recorded the locations of samples with respect to the esophageal hiatus and the gastroesophageal junction (Supplementary Tables 4–6); in rats, we also recorded the approximate distance from the gastroesophageal junction to the hepatic branch (Supplementary Table 6). The rat subdiaphragmatic vagal trunk most commonly splits into three anterior branches (hepatic, accessory celiac, and anterior gastric) and two posterior branches (celiac and posterior gastric), although variations are common (double trunks, additional branches, and different branching order) (Legros and Griffith, 1969; Prechtl and Powley, 1985). The human subdiaphragmatic vagal trunks most commonly include a single anterior trunk passing through the esophageal hiatus that splits into the hepatic and anterior gastric branches, as well as a single posterior trunk that splits into the celiac and posterior gastric branches (Tailai et al., 1980; Skandalakis et al., 1986); however, many variations were observed in terms of number of trunks passing through the esophageal hiatus, number of subdiaphragmatic branches, and locations of branches (Doubilet et al., 1948; Gravgaard, 1968; Tailai et al., 1980; Skandalakis et al., 1986).

**FIGURE 10:**
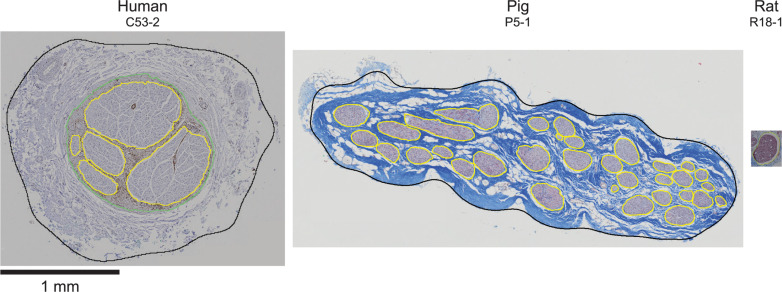
Example cervical vagus nerve cross sections from human, pig, and rat, all at the same scale.

With respect to between-species effects, the human and pig nerves were comparable sizes at each level, which would allow similar stimulation cuff electrodes to be used. However, the pig nerves had approximately ten times more fascicles and the fascicles were smaller on average—albeit overlapping with the lower range of human fascicle diameters—which would affect patterns of nerve fiber activation, since fibers in smaller fascicles have lower thresholds (Grinberg et al., 2008). Conversely, the rat nerves were approximately ten times smaller in diameter (Figure 10), and this has a substantial impact on suitable electrode designs and stimulation parameters compared to larger animals. The ratios of the mean endoneurial area of the subdiaphragmatic VN to the cervical VN were 0.20, 0.42, and 0.32 for human, pig, and rat, respectively, which is consistent with the fact that the subdiaphragmatic level of the VN has only ∼30 to 50% of the number of fibers than the cervical level (Hoffman and Schnitzlein, 1961; Mei et al., 1980; Prechtl and Powley, 1985; Asala and Bower, 1986; Soltanpour and Santer, 1996). However, this difference in number of fibers between nerve levels is not reflected in the effective nerve diameters—which informs selection of cuff electrode size—where the ratios of the mean diameter of the subdiaphragmatic VN to the cervical VN were 0.87, 0.75, and 0.68 for human, pig, and rat, respectively. In general, some of the within- and between-species differences may be due to sample-dependent durations of immersion fixation spanning 2 to 8 days (Supplement 1) and species-dependent fixation methods: the human cadavers were embalmed, the pigs were fresh, and the rats underwent intracardial perfusion of fixative, although all nerve samples were then immersion-fixed in 4% paraformaldehyde. However, the key conclusions of the study are based on larger effects than the expected effects of differences in fixation.

We compared our measurements to published data (Figure 7), although many metrics were not previously quantified. In particular, no studies quantified the fascicle diameters for human or pig VNs, and only one recent paper included nerve diameter and fascicle number for pig VN (Stakenborg et al., 2020), although their methods for quantifying nerve diameter were not provided. It should also be noted that in nerve morphology and ultrastructure studies, it is common to quantify the largest or smallest diameter, rather than effective circular diameter; we only compared to data that used the latter metric, which is more clearly defined. Our human cervical nerve diameters (1.9 ± 0.5 mm) were smaller than most prior published data, which reported mean diameters of 3.0 mm for 71 left cervical and 3.0 mm for 68 right cervical VNs across three studies (Verlinden et al., 2016; Hammer et al., 2018, 2015), but 2.0 mm for 1 left and 5 right VNs in one study (Stakenborg et al., 2020). All values are nonetheless consistent with the use of 2 mm or 3 mm inner-diameter cuff electrodes for clinical implanted cervical VNS (Santos, 2004), although VNS can alter nerve morphology with increased intra- and peri-neural connective tissue (Martín-Portugués et al., 2005). The larger diameters in literature are likely due to inconsistent removal of blood vessels and connective tissue around the nerve proper. Indeed, our number of fascicles and endoneurial cross-sectional area are consistent with the prior studies, indicating that we merely identified additional epineurial tissue. There was some subjectivity in the dissection and segmentation of the nerve boundaries for certain human samples (Figure 2). We used reproducible processes in our segmentation, although four human samples required manual adjustments to exclude extraneous tissue that was clearly not part of the nerve trunk (Figure 2). The example images in Verlinden et al. (2016) show substantial tissue beyond that which we identified as the nerve boundary, although it is unclear if it was all included in their segmentation, (Hammer et al., 2015, 2018) did not include example cross sections or image analyses, and (Stakenborg et al., 2020) did not state their image analysis methods for quantifying nerve diameter. Further, whereas (Hammer et al., 2015, 2018; Verlinden et al., 2016) all dissected intact necks, our samples were obtained following medical training courses and were therefore more likely cleared of surrounding tissues. The discrepancy may also be due to different fixation protocols: (Hammer et al., 2015, 2018) used bodies embalmed with ethanol-glycerin and immediately cast the nerves in polyvinyl siloxane, while (Verlinden et al., 2016) used femoral infusion of 10% formalin, 4 weeks with the bodies in a formalin bath, and immersion fixation in 1% OsO4 in PBS overnight. As with the removal of extraneous tissue, differences in fixation would also have greater effects on nerve diameter than on endoneurial area (given the protein content of the perineurium resulting in stronger fixation) or on number of fascicles (which should be unaffected by fixation), consistent with the observation that our nerve diameters are smaller than prior reports, but number of fascicles and endoneurial area are well-matched. Lastly, the discrepancy may reflect tissue shrinkage due to dehydration and heating, expected to be ∼15–30% (Hursh, 1939; Friede and Samorajski, 1967; Stickland, 1975; Boyd and Kalu, 1979). However, (Hammer et al., 2015, 2018) quantified the cross-sectional area using polyvinyl siloxane casts of the nerves, which are not subject to shrinkage, whereas (Verlinden et al., 2016) used paraffin embedding, and our human VN diameters were smaller than both prior reports. The effects of shrinkage on our measurements of fascicle diameter were somewhat mitigated by our focus on segmenting the perineurium rather than the endoneurium. Specifically, the endoneurium showed instances of cracking (especially for the rat samples) and whitespace between the endoneurium and inner perineurial boundary (e.g., see Methods on pig MT segmentation and Supplement 2). Conversely, the perineurium was more intact, as expected given that it contains many proteins (Pummi et al., 2004; Peltonen et al., 2013) that would be cross-linked during fixation.

Our analyzed human VN samples were from a comparable age range [54 to 90+ years old (Supplementary Table 1)] to the subjects in prior publications on human cervical VN morphology (67–103 years old) (Hammer et al., 2015, 2018; Verlinden et al., 2016), whereas VNS patients are typically younger (Milby et al., 2008). Our morphology metrics did not show trends with age for any species (Supplement 6), although our data were not collected to examine systematically the effects of aging. Studies on the effects of aging on human peripheral nerves have focused almost exclusively on somatic nerves (Spencer and Ochoa, 1981; Verdu et al., 2000), which show changes in morphology [including increased epineurial and perineurial area (Cottrell, 1940; Sladjana et al., 2008)—but no change in fascicle number or diameter (Swallow, 1966; O’Sullivan and Swallow, 1968)], ultrastructure [including decreased myelinated fiber density, decreased myelin thickness, and increased variability in the internodal length for a given fiber diameter (Lascelles and Thomas, 1966; Swallow, 1966; Ochoa and Mair, 1969; Spritz et al., 1973), with some indication of decreased unmyelinated axon diameter (Ochoa and Mair, 1969)], and electrophysiology [including slower conduction speed and smaller recorded neural signals (Mayer, 1963; Kemble, 1967; Stetson et al., 1992; Palve and Palve, 2018)]. However, a study of the human subdiaphragmatic VN—which is largely comprised of unmyelinated fibers (Pelot and Grill, 2018)—did not find any changes with age in unmyelinated fiber density, axon diameter, Schwann cell nuclei density, or number of axons per Schwann cell (Sharma and Thomas, 1973). Similarly, a study of the rat cervical VN only found small changes in the size and density of unmyelinated and myelinated fibers with age (Soltanpour and Santer, 1996), and a study of human sciatic, vagus, and sympathetic nerves indicated that the effects of aging were more pronounced for the sciatic nerve (Takahashi, 1966), although their fiber count data are unreliable as electron microscopy is needed for accurate counts of unmyelinated fibers. These ultrastructural findings are consistent with an electrophysiology study in rat VNs that found a small decrease in myelinated conduction speed with advanced age (700–900 days), but not unmyelinated conduction speed (Sato et al., 1985). The stability of the VN ultrastructure with age may be due to its position in the body that is more protected from stretching, compression, and friction as compared to somatic nerves (Sharma and Thomas, 1973; Spencer and Ochoa, 1981; Soltanpour and Santer, 1996) and its predominantly unmyelinated fiber population, since myelin is more susceptible to degeneration.

We also compared our rat VN morphology data to prior publications. The rat cervical VN was identified as monofascicular in prior studies (Fazan and Lachat, 1997; Licursi de Alcântara et al., 2008), but we observed up to six fascicles within the carotid sheath (Supplementary Figure 16). Our sample preparation was novel in that we embedded and sectioned the entire carotid sheath, rather than only sampling the nerve. We segmented and quantified the morphology of the largest fiber bundle in the sheath, consistent with prior studies; the largest cervical fascicle can be clearly identified as the VN both *in vivo* and in the micrographs, and it is the target of cuff electrodes for electrophysiology experiments. The identities of the smaller bundles in the carotid sheath are unclear; possibilities include smaller cervical vagal fascicles, vagal branches [e.g., superior laryngeal branch (Licursi de Alcântara et al., 2008)], sympathetic chain (Ding et al., 2011), hypoglossal nerve, glossopharyngeal nerve, spinal accessory nerve (Saito et al., 2018), and ansa cervicalis (Usami et al., 2013). The sizes of our rat cervical nerves were comparable to literature (Fazan and Lachat, 1997; Licursi de Alcântara et al., 2008), whereas our rat subdiaphragmatic nerves trended larger (Prechtl and Powley, 1985; Figure 7). These may reflect differences in rat strains [Sprague-Dawley, as used herein and in Prechtl and Powley (1985) vs. spontaneously hypertensive rats (SHRs) (Licursi de Alcântara et al., 2008) vs. Wistar (Fazan and Lachat, 1997)]; ages [increased nerve size from 50 to 185 days old (Fazan and Lachat, 1997), although fiber distributions and ultrastructure in Wistar rat VN did not change from 4 to 30 months (Soltanpour and Santer, 1996)]; and/or weights [mean ± SEM = 183 ± 4 g in Prechtl and Powley (1985) vs. 381 ± 38 g for our rats].

Sunderland and Bradley (1952) quantified fascicle diameter and perineurium thickness for multiple human somatic nerves (median, ulnar, radial, sciatic) that were fixed in formalin, embedded in paraffin, and stained with H&E; however, characteristics used to identify the perineurium are not defined or exemplified in figures, and the ages of the subjects were not specified (Sunderland and Bradley, 1949). Grinberg et al. (2008) analyzed these data and found that the perineurium thickness was 3.0 ± 1.0% of the fascicle diameter; a best-fit line with zero intercept of the perineurium thickness as a function of fascicle diameter had a slope of 2.6%. We re-analyzed the original (Sunderland and Bradley, 1952) data, including all 777 fascicles—whereas (Grinberg et al., 2008) only included 704 fascicles without specifying their exclusions—and identified the slope of the best-fit line with zero intercept as 2.75% (*R*^2^ = 0.51) (Figures 9C,D). While we found that the rat perineurium thickness was similar to this published relationship, albeit slightly thicker for smaller fascicle diameters and thinner for larger fascicle diameters, the human and pig perineurium was substantially thicker, especially for smaller fascicles (Figure 9B). The (Sunderland and Bradley, 1952) ratio of perineurium thickness to fascicle diameter also trended toward larger values for smaller fascicles in their raw data, but to a lesser extent than seen in our VNs (Figures 9D vs. B). This discrepancy could reflect morphological differences between somatic and autonomic nerves, differences in identification methods for defining the perineurial boundaries, or age-dependent effects. Regardless, the relationship of thk_peri_ = 3%^∗^*d*_fasc_ has been used in several computational models of compound peripheral nerves (Grinberg et al., 2008; Schiefer et al., 2008; Kent and Grill, 2013; Raspopovic et al., 2017; Pelot et al., 2019), whereas our data show that human and pig perineurium is thicker, and this will substantially affect modeled activation and block thresholds (Grinberg et al., 2008; Pelot et al., 2019) given the high resistivity of the perineurial tissue (Weerasuriya et al., 1984).

The sex of our subjects is provided in Supplement 1. There do not appear to be differences in VN morphology between sides or sexes in prior literature, except for a larger posterior nerve at the subdiaphragmatic level in humans (Gravgaard, 1968; Tailai et al., 1980). Prior studies did not detect side or sex differences in human cervical VN nerve size [(Hammer et al., 2015) used 20 female left, 19 female right, 13 male right, and 14 male left samples; (Verlinden et al., 2016) used 11 left and 11 right samples, without specifying sex] or number of fascicles [(Seki et al., 2014) samples outlined below; (Verlinden et al., 2016) samples outlined above; (Hammer et al., 2018) used 26 left and 25 right samples across 16 females and 11 males]. (Seki et al., 2014) used 28 left cervical, 29 right cervical, 24 left thoracic, and 23 right thoracic samples from 11 females and 20 males; they did not detect effects of side, sex, level (cervical vs. thoracic) or ethnicity on the number of fascicles or endoneurial cross-sectional area, although (Verlinden et al., 2016) found larger endoneurial cross-sectional area on the right side. (Licursi de Alcântara et al., 2008) collected left and right VN samples at the rostral and caudal cervical levels for 5 female and 5 male rats; they did not find effects of side, level (rostral cervical vs. caudal cervical), or sex on nerve size, except for smaller nerve area on the right side for the distal portion of the cervical VN for female samples, despite significant sex differences in arterial pressure and heart rate. For the rat subdiaphragmatic VN, (Prechtl and Powley, 1985) did not find sex differences (7F/15M) in gross anatomy or morphology. A larger sample size would be required to assess differences in sex in our data, particularly for humans given their greater biological variability; the raw data in Figure 6 do not suggest an effect of sex on morphology. Conversely, there do appear to be differences in vagal fibers between sexes with regards to ion channels (Sturdy et al., 2017) and axonal ultrastructure (Dollé et al., 2018).

We quantified the morphology of the VN in humans, pigs, and rats at the cervical and subdiaphragmatic levels, including nerve size, fascicle size, number of fascicles, proportions of tissue types, and perineurium thickness. Human and pig nerves were similar sizes at both the cervical and subdiaphragmatic levels, indicating that clinical cuff electrodes will fit on the pig large animal model, but given that pigs have ten times more fascicles which are smaller than human fascicles, their fibers are expected to have lower activation thresholds. Conversely, rat nerves are ten times smaller in diameter and generally monofascicular, and therefore require much smaller cuff electrodes and will have lower thresholds given smaller electrode-fiber distances. Further, while rat perineurium thickness was comparable to the established 3% of fascicle diameter, pig and human perineurium was thicker, which will substantially increase thresholds in computational models. This novel compendium of quantitative VN morphology is critical for seeding computational models of VNS, for understanding variability in therapeutic response across individuals, and for translating preclinical findings to the clinical target to advance therapeutic designs and efficacy of VNS.

## Data Availability Statement

The data associated with this study are publicly available on the NIH SPARC Portal (RRID:SCR_017041) at Pelot et al. (2020a, b, c, d, e, f, g).

## Author Contributions

NP and WG conceived of and designed the project. NP, GG, and EM collected the nerve samples. NP and JE designed the histology protocols. KC and JE conducted the histology. NP, GG, and JC imaged the slides and segmented the images. NP conducted all the data analyses and wrote the manuscript. All authors revised and approved the manuscript.

## Conflict of Interest

The authors declare that the research was conducted in the absence of any commercial or financial relationships that could be construed as a potential conflict of interest.
