# Supplementary material for: Quantified Morphology of the Cervical and Subdiaphragmatic Vagus Nerves of Human, Pig, and Rat
Source: Front Neurosci. 2020 Nov 4;14:601479. doi: 10.3389/fnins.2020.601479 (PMC7672126; doi:10.3389/fnins.2020.601479)
Supplement: Supplementary file 1 [file Data_Sheet_1.pdf]

Quantified Morphology of the Cervical and Subdiaphragmatic Vagus Nerves of Human, Pig, and Rat

Supplemental Materials

Nicole A. Pelot[1*], Gabriel B. Goldhagen[1], Jake E. Cariello[1], Eric D. Musselman[1], Kara A. Clissold[2], J. Ashley Ezzell[2], Warren M. Grill[1,3,4,5*]

[1]Duke University, Department of Biomedical Engineering, Durham, NC, USA
[2]University of North Carolina, Histology Research Core, Chapel Hill, NC, USA
[3]Duke University, Department of Electrical and Computer Engineering, Durham, NC, USA
[4]Duke University, Department of Neurobiology, Durham, NC, USA
[5]Duke University, Department of Neurosurgery, Durham, NC, USA

*** Correspondence:**
Nicole A. Pelot
nikki.pelot@duke.edu
Warren M. Grill
warren.grill@duke.edu

doi: 10.3389/fnins.2020.601479

1 Supplement 1

1.1 Subject Metadata

Table 1. Metadata for human cadaveric subjects. “Analyzed morphology” indicates whether the vagus nerve morphology of at least one sample from that cadaver was quantified. Ages above 89 years old were recorded as “90+” because specifying the specific numbers constitutes protected health information.

Analyzed morphology	Subject ID	Age (years)	Sex	Body mass (lb)	Height (inches)	Days from death to embalming	Cause of death	Other diseases	Race
--	C42	61	Male	140	69	3 to 4	Sever emphysema	--	Unknown
--	C43	76	Male	192	68	3 to 4	Bladder cancer	--	Unknown
--	C44	59	Female	87	62	3 to 4	Lung cancer	--	Unknown
--	C45	89	Male	127	69	3 to 4	Parkinson's disease	--	Unknown
Y	C46	90+	Female	130	67	3 to 4	CHF	--	Unknown
Y	C47	78	Female	130	63	3 to 4	CHF	Hyperlipidemia	Unknown
--	C48	90	Male	179	68	3 to 4	Prostate cancer	Hypertension, Diabetes	Unknown
--	C49	89	Female	100	59	3 to 4	Malnutrition	--	Unknown
Y	C50	67	Male	175	67	3 to 4	Respiratory failure	--	Unknown
--	C51	73	Female	149	67	2	Complications of wrist fracture and Alzheimer's	--	Unknown
Y	C53	84	Male	160	67	1	Squamous cell carcinoma of skin	Malignant neoplasma of left & right lung, laryngeal cancer, hypertension	Unknown
Y	C54	90+	Male	130	70	3	Metastatic prostate cancer	History of CVA, stage 3 kidney disease	Unknown
Y	C55	90+	Male	134	73	2	Idiopathic pulmonary fibrosis	Pulmonary arthrosis, osteoporosis, RA, b/l hernia, HX spinal compression FX, dyspepsia	Caucasian
Y	C56	59	Female	123	66	4	Respiratory failure	Non-small cell lung cancer, chronic obstruction pulmonary	Caucasian
Y	C57	90+	Male	192	72	2	Coronary artery disease	Hypertension, chronic respiratory failure, systolic dysfunction, pleural effusion	Caucasian
Y	C58	86	Female	148		4	Hypertensive heart disease with heart failure	Heart issues, hyperlipidemia, essential (primary) hypertension, chronic atrial fibrillation, hematoma in stomach	Unknown
--	C59	81	Female	104	61	2	Vascular dementia	Cardiovascular disease, hyper tension, stroke, chronic kidney disease	Unknown
Y	C60	90+	Female	90	64	1	Urinary tract infection	--	Unknown
--	C61	88	Female	152	65	2	Liver cancer	Colon cancer, asthma, had pacemaker	Unknown
--	C62	79	Male	127	66	0	Esophageal cancer	Severe scoliosis, bowel obstruction, had spinal stimulator	Unknown
Y	C63	85	Female	145	65	1 to 4	Myocardial infarction	--	Caucasian
Y	C64	56	Male	150	70	1 to 4	Cardiac arrest	Spinal fusion	Caucasian

Y	C65	54	Female	122	59	1 to 4	Kidney cancer	--	Caucasian
--	C66	75	Male	112	67	1 to 4	COPD	--	Caucasian
--	C67	90+	Female	114	62	1 to 4	Congestive heart failure	Alzheimer's	Unknown
--	C68	89	Male	150	65	1 to 4	Anoxic brain injury	Firm white gall bladder (no bile); descending aorta blockage, leadless pacemaker	Caucasian
Y	C69	87	Male	130	70	1 to 4	Pleural effusion	--	Caucasian
Y	C70	84	Male	149	70	1 to 4	Lung cancer	Macular degeneration	Unknown
--	C71	90+	Female	126	63	1 to 4	Dementia	--	Caucasian
--	C72	79	Female	165	64	1 to 4	Interstitial lung disease	Recent stroke, dementia	Caucasian

Table 2. Metadata for pigs. "Analyzed morphology" indicates whether the complete vagus nerve morphology of at least one sample from that pig was quantified. "Analyzed perineurium" indicates whether the perineurium thickness of at least one sample from that pig was quantified using anti-fibronectin immunofluorescence.

**Analyzed morphology**	**Analyzed perineurium**	**Subject ID**	**Age (weeks)**	**Sex**	**Body mass (kg)**	**Days from sample collection to embedding in paraffin**	**Use/treatment prior to post-mortem dissection**
Y	--	P4	13	Female	34.3	7	Laparotomy training
Y	--	P5	13	Female	32.8	7	Laparotomy training
Y	--	P6	13	Female	28.2	7	Laparotomy training
--	--	P7	13	Female	29.6	7	Laparotomy training
Y	--	P8	13	Female	28.7	7	Laparotomy training
Y	Y	P9	15	Female	45	5	Laparotomy training
Y	--	P10	15	Female	45	5	Laparotomy training
Y	Y	P11	10.5	Male	24.4	5	Heparinized & perfused with saline (6L) + heparin (10000 units per 10 mL / 1L saline)
Y	Y	P12	10.5	Male	23.4	5	4 days with paclitaxel (chemo drug); 0.5 mL/kg each day (clinical dosage); Heparinized & perfused with saline (5L) + heparin (10000 units per 10 mL / 1L saline)
Y	Y	P13	10.5	Male	23.8	5	4 days with paclitaxel (chemo drug); 0.5 mL/kg each day (clinical dosage) + stem cell treatment in intrathecal space (500000 bone marrow strombal cells in 5 mL of saline) 1 day before euthanasia; Heparinized & perfused with saline (5L) + heparin (10000 units per 10 mL / 1L saline)
Y	Y	P14	14	Female	38.5	5	Laparotomy training
Y	Y	P15	14	Female	39.5	5	Laparotomy training

Table 3. Metadata for rats. "Analyzed morphology" indicates whether the vagus nerve morphology of at least one sample from that rat was quantified. Note 1: Only date of arrival, not date of birth, was available. Given that a 250 g female rat was ordered, it was assumed to be 15 weeks old upon arrival based on the weight vs. age data from Charles River: https://www.criver.com/products-services/find-model/sas-sprague-dawley-rat?region=3611. Note 2: Only date of arrival, not date of birth, was available. Given that a 250 g male rat was ordered, it was assumed to be 8 weeks old upon arrival based on the weight vs. age data from Charles River: https://www.criver.com/products-services/find-model/sas-sprague-dawley-rat?region=3611.

**Analyzed morphology**	**Subject ID**	**Age (days)**	**Sex**	**Body mass (g)**	**Days from sample collection to embedding in paraffin**
Y	R16	94	Male	534	8
Y	R17	189	Male	422	8
Y	R18	268	Female	312	8
Y	R20	75	Male	431	6
Y	R21	115	Female(Note 1)	266	6
Y	R22	114	Female(Note 1)	260	7
Y	R23	88	Male	466	7
Y	R24	103	Female	292	8
Y	R25	141	Male(Note 2)	570	6
Y	R26	89	Female	258	8

1.2 Sample Metadata

Table 4. Metadata for human cadaveric vagus nerve samples, all of which have Masson's trichrome-stained cross sections. "Analyzed morphology" indicates samples that underwent anti-claudin-1 immunohistochemistry for which the micrographs were then segmented and analyzed to quantify the morphology, as well as the cervical and subdiaphragmatic no primary controls (ctl). The "Distance from…" columns provide the distance from the center of the sample (from which we sectioned) to the indicated landmark. Note 1: This cervical vagus nerve had two trunks. Sample C55-3 was medial to sample C55-4. Note 2: We assumed that the embalming occurred 3 days after death. See Table 1 for range of delays between death and embalming. GE: gastroesophageal.

**Analyzed morphology**	**Subject ID**	**Sample ID**	**Level**	**Laterality**	**Distance from carotid bifurcation (mm)**	**Distance from esophageal hiatus and from GE junction (mm)**	**Days from embalming to sample collection**	**Days from sample collection to embedding in paraffin**
--	C42	2	Cervical	Left	15	--	438 (Note 2)	2
--	C42	1	Cervical	Right	20	--	438 (Note 2)	2
--	C43	2	Cervical	Left	25	--	427 (Note 2)	2
--	C43	1	Cervical	Right	25	--	427 (Note 2)	2
--	C44	2	Cervical	Left	15	--	218 (Note 2)	2
--	C44	1	Cervical	Right	15	--	218 (Note 2)	2
--	C45	2	Cervical	Left	15	--	486 (Note 2)	2
--	C45	1	Cervical	Right	25	--	486 (Note 2)	2
Y	C46	2	Cervical	Left	30	--	365 (Note 2)	2
--	C46	1	Cervical	Right	25	--	365 (Note 2)	2
Y	C47	2	Cervical	Left	30	--	393 (Note 2)	2
--	C47	1	Cervical	Right	35	--	393 (Note 2)	2
--	C48	1	Cervical	Right	20	--	447 (Note 2)	2
--	C49	1	Cervical	Right	30	--	389 (Note 2)	2
Y	C50	2	Cervical	Left	30	--	445	2
--	C50	1	Cervical	Right	30	--	445	2
--	C51	1	Subdiaphragmatic	Anterior	--	7	192	5
--	C51	3	Subdiaphragmatic	Posterior	--	10	192	5
--	C53	3	Subdiaphragmatic	Anterior	--	10	337	7
Y	C53	2	Cervical	Left	40	--	337	7
--	C53	4	Subdiaphragmatic	Posterior	--	10	337	7
--	C53	1	Cervical	Right	30	--	337	7
Y	C54	2	Subdiaphragmatic	Anterior	--	12	368	5
Y	C54	3	Cervical	Left	40	--	402	6
--	C54	1	Subdiaphragmatic	Posterior	--	12	368	5
--	C54	4	Cervical	Right	45	--	402	6
ctl	C55	1	Subdiaphragmatic	Anterior	--	10	294	5

--	C55	4	Cervical	Left[Note 1]	40	--	328	6
Y	C55	3	Cervical	Left[Note 1]	40	--	328	6
--	C55	2	Subdiaphragmatic	Posterior	--	10	294	5
--	C55	5	Cervical	Right	35	--	328	6
Y	C56	1	Subdiaphragmatic	Anterior	--	12	230	5
Y	C56	3	Cervical	Left	20	--	264	6
--	C56	2	Subdiaphragmatic	Posterior	--	10	230	5
--	C56	4	Cervical	Right	30	--	264	6
Y	C57	1	Subdiaphragmatic	Anterior	--	12	290	5
Y	C57	3	Cervical	Left	40	--	324	6
--	C57	2	Subdiaphragmatic	Posterior	--	10	290	5
--	C57	4	Cervical	Right	30	--	324	6
Y	C58	1	Subdiaphragmatic	Anterior	--	10	332	5
Y	C58	3	Cervical	Left	40	--	366	6
--	C58	2	Subdiaphragmatic	Posterior	--	12	332	5
--	C58	4	Cervical	Right	30	--	366	6
--	C59	3	Cervical	Left	40	--	387	6
--	C59	4	Cervical	Right	35	--	387	6
ctl	C60	3	Cervical	Left	35	--	282	6
--	C60	4	Cervical	Right	30	--	282	6
--	C61	1	Cervical	Left	40	--	400	6
--	C61	2	Cervical	Right	30	--	400	6
--	C62	1	Cervical	Left	30	--	357	6
--	C62	2	Cervical	Right	35	--	357	6
Y	C63	1	Subdiaphragmatic	Anterior	--	10	370[Note 2]	8
Y	C64	1	Subdiaphragmatic	Anterior	--	10	368[Note 2]	8
--	C64	2	Subdiaphragmatic	Posterior	--	12	368[Note 2]	8
Y	C65	1	Subdiaphragmatic	Anterior	--	10	402[Note 2]	8
--	C65	2	Subdiaphragmatic	Posterior	--	10	402[Note 2]	8
--	C66	2	Subdiaphragmatic	Posterior	--	12	162[Note 2]	8
--	C67	1	Subdiaphragmatic	Anterior	--	10	297[Note 2]	8
--	C67	2	Subdiaphragmatic	Posterior	--	10	297[Note 2]	8
--	C68	1	Subdiaphragmatic	Anterior	--	10	568[Note 2]	8
--	C68	2	Subdiaphragmatic	Posterior	--	10	568[Note 2]	8
Y	C69	1	Subdiaphragmatic	Anterior	--	10	133[Note 2]	8
--	C69	2	Subdiaphragmatic	Posterior	--	10	133[Note 2]	8
Y	C70	1	Subdiaphragmatic	Anterior	--	10	150[Note 2]	8
--	C70	2	Subdiaphragmatic	Posterior	--	10	150[Note 2]	8
--	C71	1	Subdiaphragmatic	Anterior	--	10	330[Note 2]	8

--	C71	2	Subdiaphragmatic	Posterior	--	12	330(Note 2)	8
--	C72	1	Subdiaphragmatic	Anterior	--	10	403(Note 2)	8
--	C72	2	Subdiaphragmatic	Posterior	--	10	403(Note 2)	8

Table 5. Metadata for pig vagus nerve samples, all of which have Masson's trichrome-stained cross sections (except P7-7). (A) Samples for which the Masson's trichrome cross section was segmented and analyzed to quantify the morphology. (B) Samples that underwent anti-fibronectin immunofluorescence, including the cervical and subdiaphragmatic controls (ctl; no primary and no primary/no secondary antibody controls for each control sample). (C) Samples for which the perineurium in the anti-fibronectin immunofluorescence cross section was segmented and analyzed to quantify perineurium thickness. The "Distance from carotid bifurcation" and "Distance from esophageal…" columns provide the distance from the center of the sample (from which we sectioned) to the indicated landmark. See Methods for the description of the "Distance from bottom of jaw to top of sternum". GE: gastroesophageal.

(A)	(B)	(C)	Subject ID	Sample ID	Level	Laterality	Distance from carotid bifurcation (cm)	Distance from bottom of jaw to top of sternum (cm)	Distance from esophageal hiatus and from GE junction (mm)	Time from death to collection (hr)
--	--	--	P4	2	Cervical	Right	Not recorded	Not recorded	--	1.75
Y	Y	--	P4	3	Subdiaphragmatic	Anterior	--	--	15	2.5
--	--	--	P4	4	Subdiaphragmatic	Posterior	--	--	20	2.5
Y	Y	--	P5	1	Cervical	Left	Not recorded	Not recorded	--	2.25
--	--	--	P5	2	Cervical	Right	Not recorded	Not recorded	--	2.75
Y	Y	--	P5	3	Subdiaphragmatic	Anterior	--	--	20	3
--	--	--	P5	4	Subdiaphragmatic	Posterior	--	--	25	3
--	ctl	--	P6	1	Subdiaphragmatic	Anterior	--	--	20	1
--	--	--	P6	3	Subdiaphragmatic	Posterior	--	--	22.5	1
--	--	--	P6	5	Cervical	Right	5.25	13	--	2
Y	Y	--	P6	7	Cervical	Left	5	13	--	2.5
--	--	--	P7	1	Subdiaphragmatic	Anterior	--	--	15	2
--	--	--	P7	3	Subdiaphragmatic	Posterior	--	--	25	2
--	--	--	P7	5	Cervical	Right	6.75	13	--	3.5
--	ctl	--	P7	7	Cervical	Left	6.5	13	--	3.5
Y	Y	--	P8	1	Subdiaphragmatic	Anterior	--	--	25	4
--	--	--	P8	3	Subdiaphragmatic	Posterior	--	--	35	4
--	--	--	P8	5	Cervical	Right	5	13	--	4
Y	Y	--	P8	7	Cervical	Left	4.25	13	--	4
--	--	--	P9	1	Subdiaphragmatic	Anterior	--	--	22.5	1

--	--	--	P9	2	Subdiaphragmatic	Posterior	--	--	35	1
Y	Y	Y	P9	3	Cervical	Left	6.25	14	--	1.25
--	--	--	P9	4	Cervical	Right	7.25	14	--	1.5
Y	Y	--	P10	1	Subdiaphragmatic	Anterior	--	--	25	1
--	--	--	P10	2	Subdiaphragmatic	Posterior	--	--	30	1
--	--	--	P10	4	Cervical	Right	9	15	--	1.25
Y	Y	Y	P11	1	Cervical	Left	6	15	--	3
--	--	--	P11	2	Cervical	Right	6	15	--	3
Y	Y	--	P11	3	Subdiaphragmatic	Anterior	--	--	25	3
--	--	--	P11	4	Subdiaphragmatic	Posterior	--	--	25	3
Y	Y	Y	P12	1	Cervical	Left	4.75	13	--	1.5
--	--	--	P12	2	Cervical	Right	5	13	--	1.5
Y	Y	Y	P12	3	Subdiaphragmatic	Anterior	--	--	27.5	1.5
--	--	--	P12	4	Subdiaphragmatic	Posterior	--	--	27.5	1.5
Y	Y	--	P13	1	Cervical	Left	6	13	--	1.5
--	--	--	P13	2	Cervical	Right	6	13	--	1.5
Y	Y	Y	P13	3	Subdiaphragmatic	Anterior	--	--	25	1.5
--	--	--	P13	4	Subdiaphragmatic	Posterior	--	--	25	1.5
--	--	--	P14	1	Cervical	Right	7.9	15	--	1
Y	Y	Y	P14	2	Cervical	Left	8	15	--	1
Y	Y	Y	P14	3	Subdiaphragmatic	Anterior	--	--	25	2.5
--	--	--	P14	4	Subdiaphragmatic	Posterior	--	--	25	2.5
--	--	--	P15	1	Cervical	Right	6	16	--	1.5
Y	Y	--	P15	2	Cervical	Left	7.25	16	--	1.5
Y	Y	Y	P15	3	Subdiaphragmatic	Anterior	--	--	20	2
--	--	--	P15	4	Subdiaphragmatic	Posterior	--	--	20	2

Table 6. Metadata for rat vagus nerve samples, all of which have Masson's trichrome-stained cross sections. "Analyzed morphology" indicates samples that underwent segmentation to quantify the morphology. "Laterality" is "Ant & post" (anterior and posterior) for the subdiaphragmatic samples because we dissected and sampled the whole esophagus with the vagus nerves attached. The "Distance from carotid bifurcation" and "Distance from esophageal hiatus and from GE junction" columns provide the distance from the center of the sample (from which we sectioned) to the indicated landmark. GE: gastroesophageal.

Analyzed morphology	Subject ID	Sample ID	Level	Laterality	Distance from carotid bifurcation (mm)	Distance from esophageal hiatus and from GE junction (mm)	Distance from esophageal hiatus to hepatic branch (mm)
Y	R16	1	Subdiaphragmatic	Ant & post	--	6.5	4
--	R16	2	Cervical	Right	12	--	--
Y	R16	3	Cervical	Left	10.5	--	--
Y	R17	1	Subdiaphragmatic	Ant & post	--	9	7
--	R17	2	Cervical	Right	11	--	--
Y	R17	3	Cervical	Left	10.5	--	--
Y	R18	1	Cervical	Left	13.5	--	--
--	R18	2	Cervical	Right	11.5	--	--
Y	R20	1	Subdiaphragmatic	Ant & post	--	11.5	11
--	R20	2	Cervical	Right	12	--	--
Y	R20	3	Cervical	Left	10.5	--	--
Y	R21	1	Subdiaphragmatic	Ant & post	--	10	10
--	R21	2	Cervical	Right	13	--	--
Y	R21	3	Cervical	Left	12	--	--
Y	R22	1	Subdiaphragmatic	Ant & post	--	7.5	6
--	R22	2	Cervical	Right	12	--	--
Y	R22	3	Cervical	Left	12	--	--
Y	R23	1	Subdiaphragmatic	Ant & post	--	11	12
--	R23	2	Cervical	Right	10.5	--	--
Y	R23	3	Cervical	Left	11	--	--
Y	R24	1	Subdiaphragmatic	Ant & post	--	10	10
--	R24	2	Cervical	Right	10	--	--
Y	R24	3	Cervical	Left	9	--	--
Y	R25	1	Subdiaphragmatic	Ant & post	--	10	11
--	R25	2	Cervical	Right	10.5	--	--
Y	R25	3	Cervical	Left	14.5	--	--
Y	R26	1	Subdiaphragmatic	Ant & post	--	8.5	10

2 Supplement 2

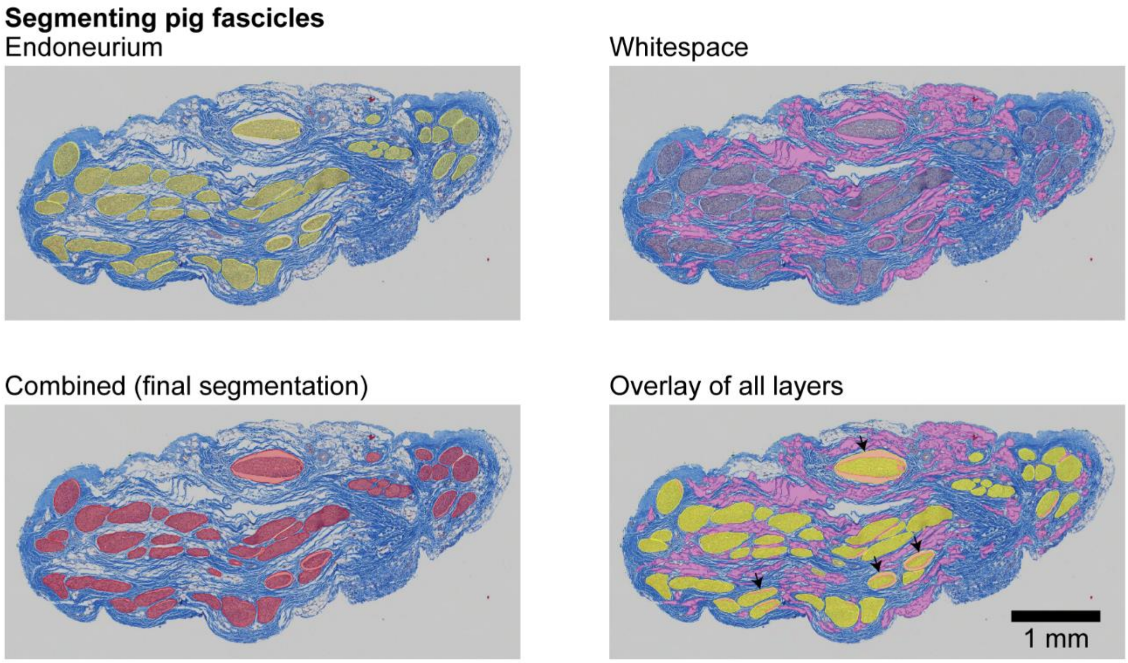

Figure 11. Example of pig fascicle segmentation. Top left: Endoneurium in yellow. Top right: Whitespace, meeting certain criteria (e.g., circularity), in pink. Bottom left: Final fascicle segmentation in red, where whitespace adjacent to endoneurium and endoneurium proper are merged. Bottom right: Overlay of all layers, where arrowheads provide examples of orange areas where the whitespace filled in the fascicular area to the inner edge of the perineurium.

3 Supplement 3

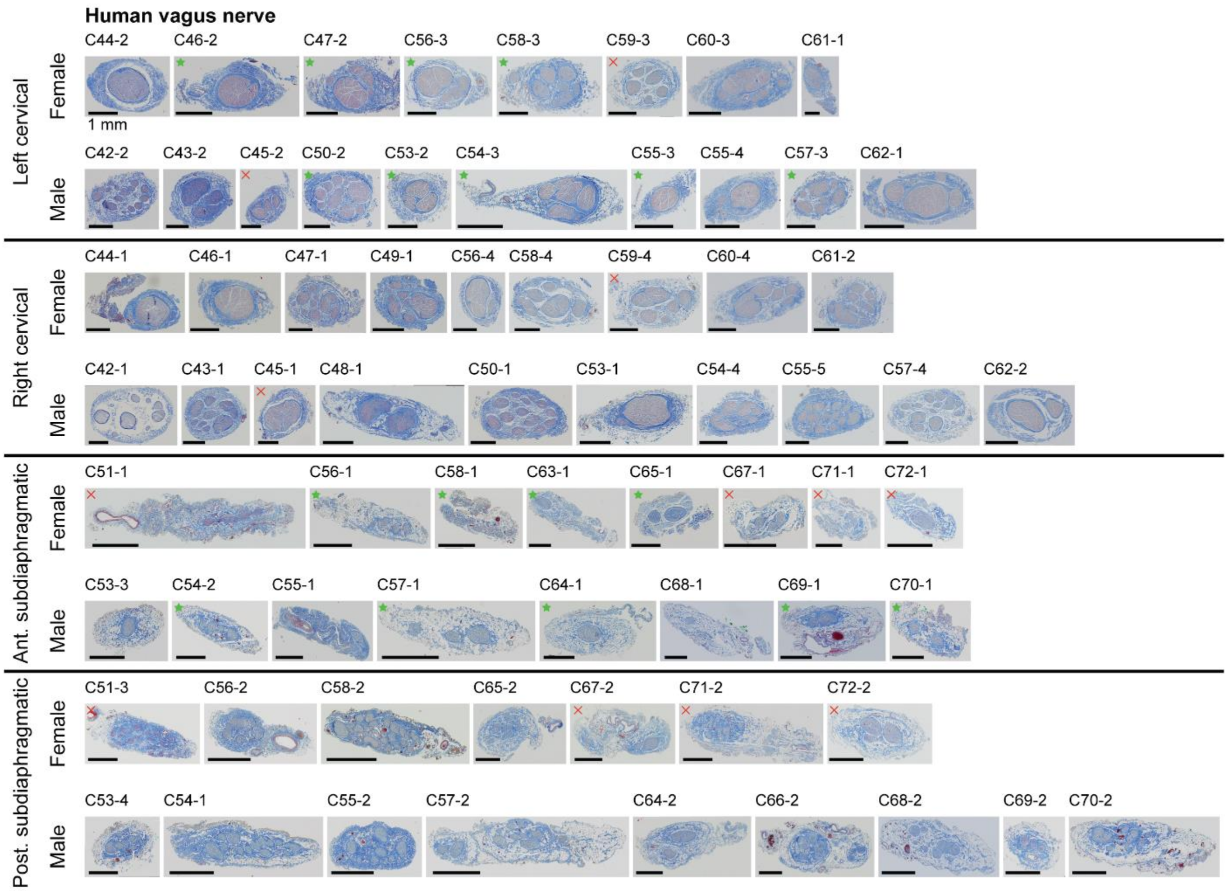

Figure 12. Human vagus nerve samples stained with Masson's trichrome. The green stars indicate the samples for which we quantified the morphology. The red X's indicate the samples from subjects with known neuropathies that were excluded when selecting samples for morphological analyses. Specifically, C45 had Parkinson's disease, C59 had vascular dementia, C51 and C67 had Alzheimer's, and C71 and C72 had dementia. All scale bars are 1 mm long. All original micrographs are available at (Pelot et al., 2020c).

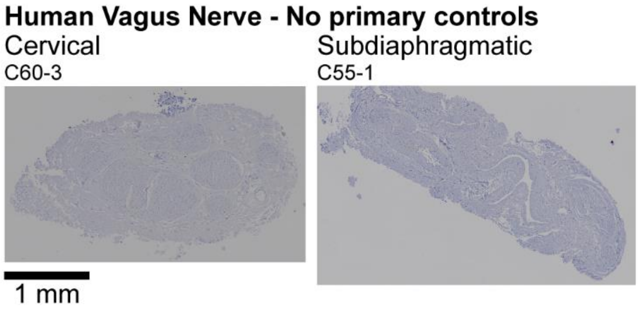

Figure 13. No primary controls for human vagus nerve samples that underwent claudin-1 immunohistochemistry. The original micrographs are available at (Pelot et al., 2020a).

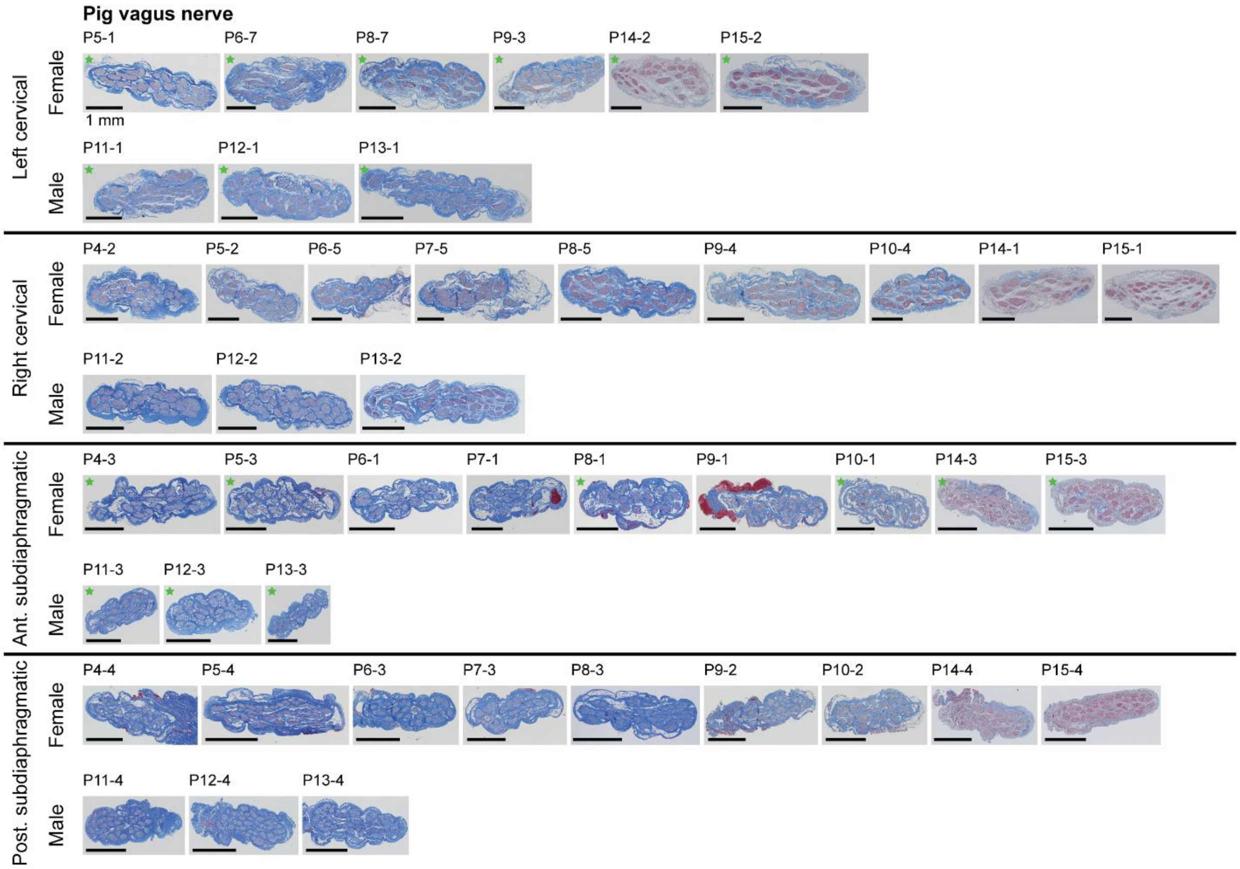

Figure 14. Pig vagus nerve samples stained with Masson's trichrome. The green stars indicate the samples for which we quantified the morphology. All scale bars are 1 mm long. All original micrographs are available at (Pelot et al., 2020d).

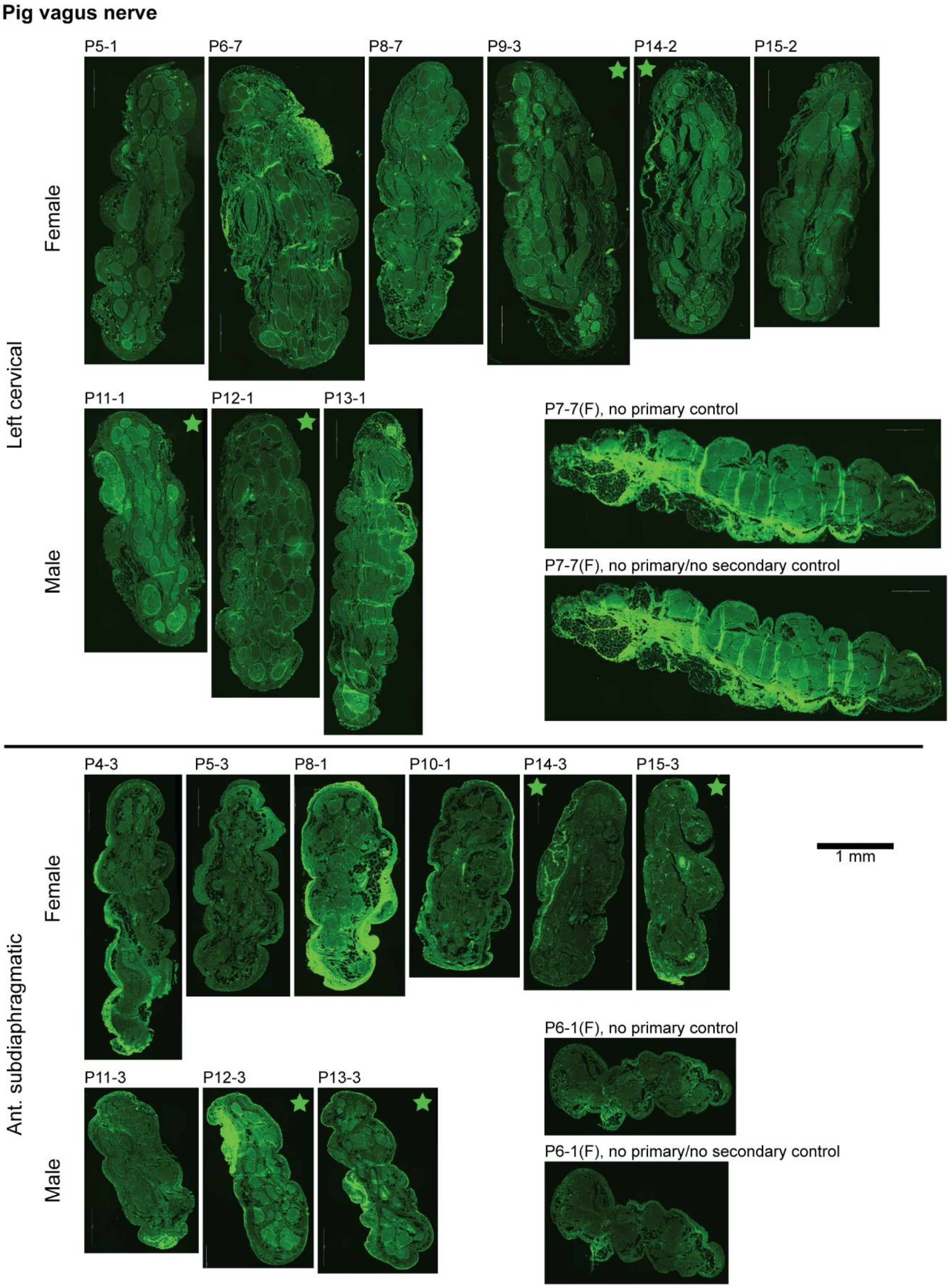

Figure 15. Pig vagus nerve samples that underwent anti-fibronectin immunofluorescence. The green stars indicate the samples for which we quantified the perineurium thickness. All images correspond to the same 1 mm scale bar. All original micrographs are available at (Pelot et al., 2020b).

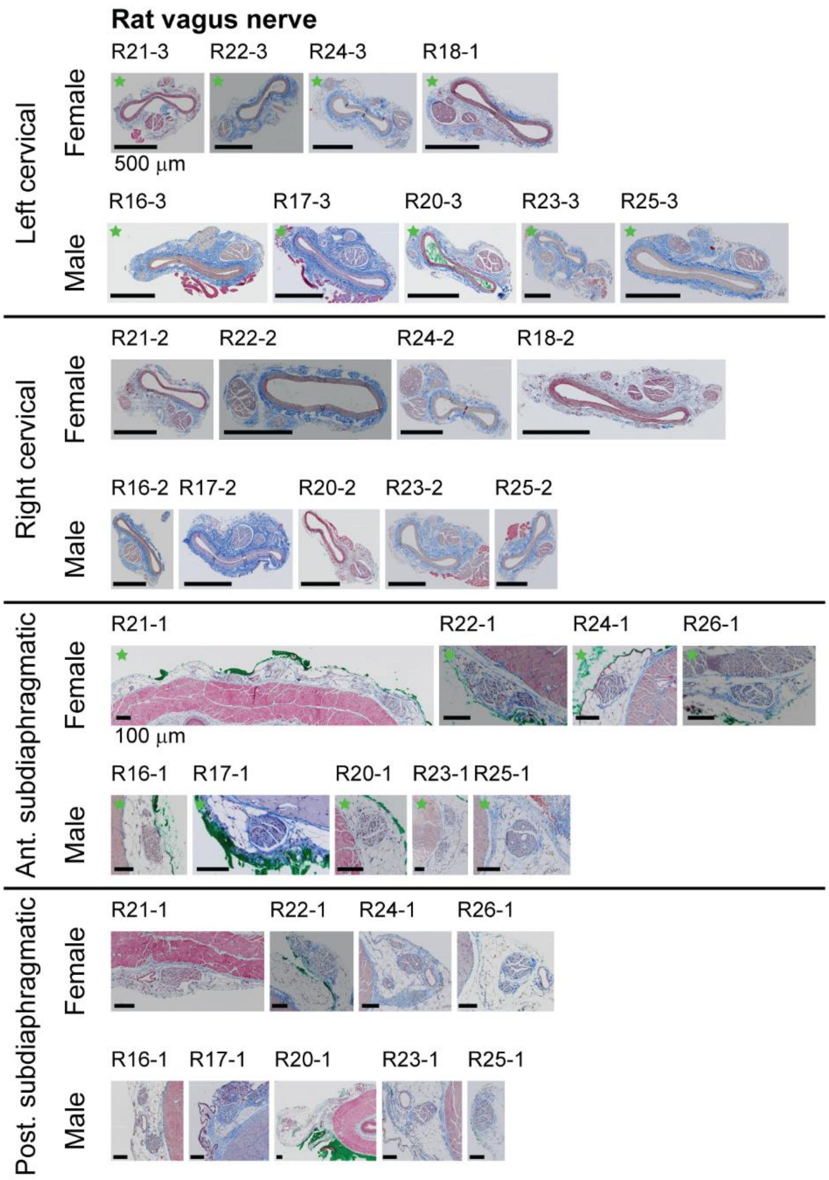

Figure 16. Rat vagus nerve samples stained with Masson's trichrome. The green stars indicate the samples for which we quantified the morphology. In addition to the cross sections of the vagus nerve, the cervical micrographs also show the common carotid artery, as well as other nerves in the carotid sheath, while the subdiaphragmatic micrographs show the edge of the esophagus. All cervical scale bars are 0.5 mm long (top half) and all subdiaphragmatic scale bar are 0.1 mm long (bottom half). All original micrographs are available at (Pelot et al., 2020e).

4 Supplement 4

Vagus Nerve Morphology: Summary Statistics and Literature Review

See Excel spreadsheet with summary statistics for our data, as well as a literature review of VN morphological parameters.

5 Supplement 5

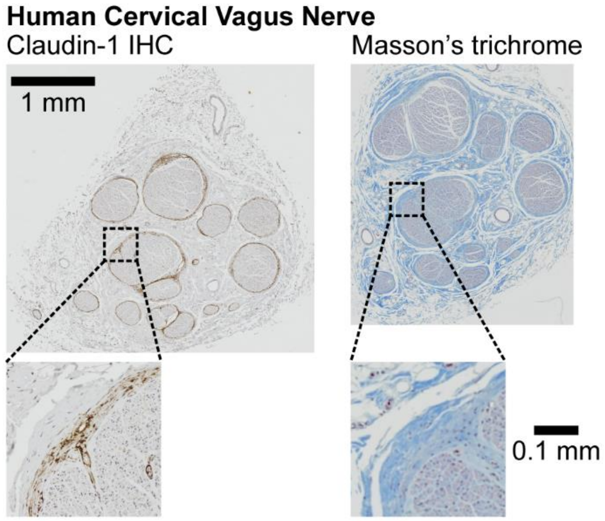

Figure 17. Comparison of human perineurium segmentation with claudin-1 IHC (left) versus Masson's trichrome staining (right). These sections are from the same sample, but the fascicular organization can be seen to change over the small distance (~250 µm). The zoomed views show a matched location between the two images where the Masson's trichrome suggested a thicker perineurium, but the claudin-1 more selectively identified the perineural tissue.

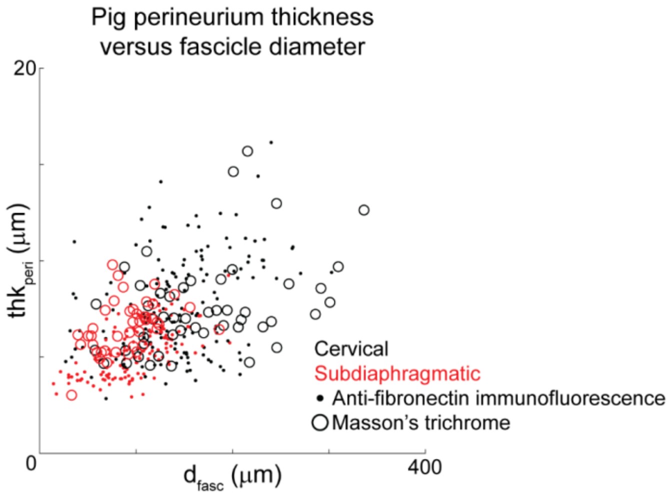

Figure 18. Perineurium thickness as a function of effective fascicle diameter for pig vagus nerve samples stained with Masson's trichrome (open circles; one cervical and one subdiaphragmatic) or labeled with anti-fibronectin (dots; four cervical and four subdiaphragmatic).

6 Supplement 6

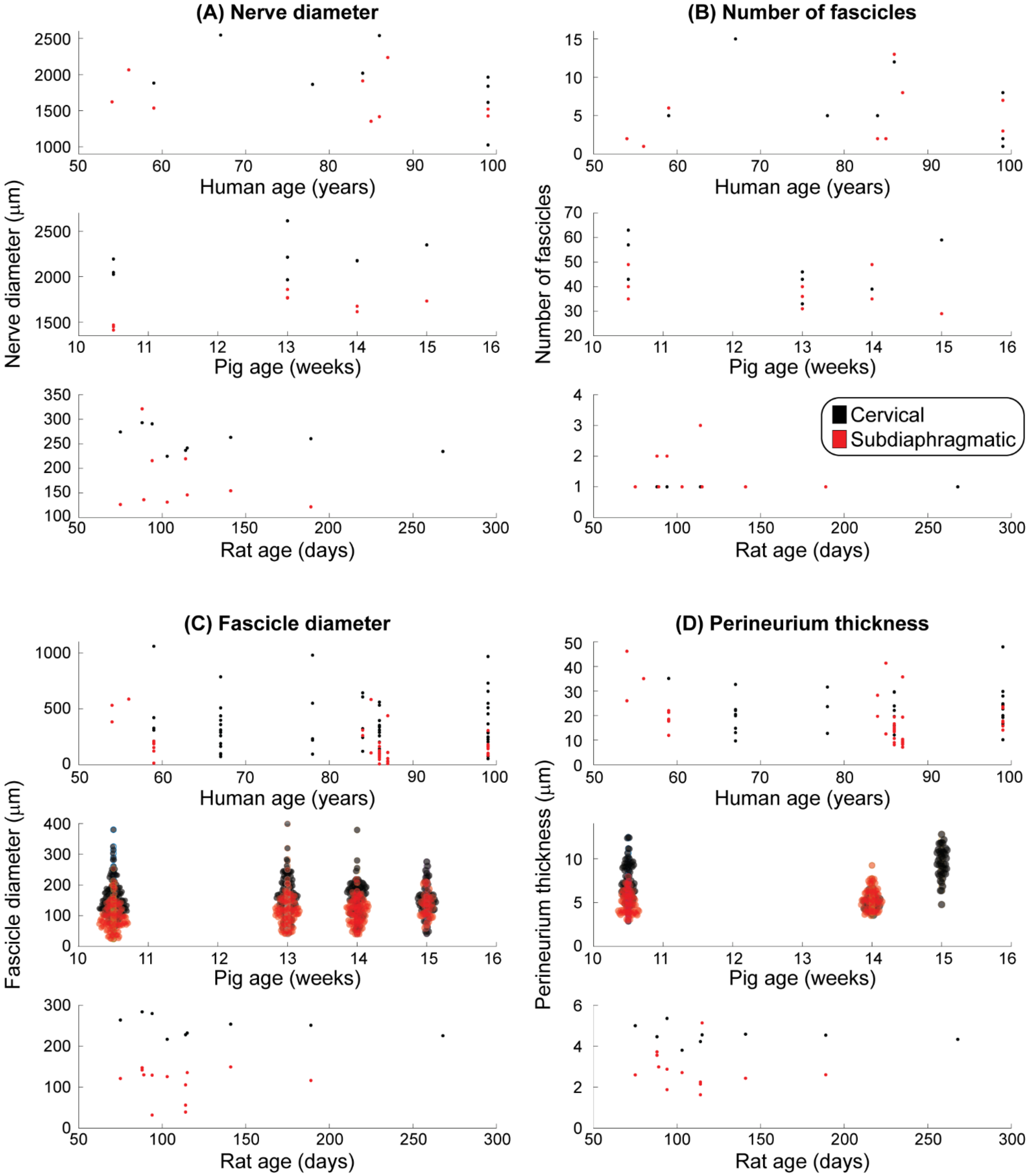

Figure 19. Nerve morphology metrics versus age. Note that all human subjects over 89 years old were plotted as 99 years old.

References

Pelot, N.A., Ezzell, J.A., Goldhagen, G.B., Cariello, J.E., Clissold, K.A., Grill, W.M., 2020a. Quantified Morphology of the Human Vagus Nerve with Anti-Claudin-1. Version 6, SPARC Data & Resource Center. https://doi.org/10.26275/nluu-1ews

Pelot, N.A., Ezzell, J.A., Goldhagen, G.B., Clissold, K.A., Grill, W.M., 2020b. Quantified Morphology of the Pig Vagus Nerve with Anti-Fibronectin. Version 1, SPARC Data & Resource Center. https://doi.org/10.26275/8pc2-rhu2

Pelot, N.A., Ezzell, J.A., Goldhagen, G.B., Musselman, E.D., Cariello, J.E., Clissold, K.A., Grill, W.M., 2020c. Human Vagus Nerve Stained with Masson's Trichrome. Version 3, SPARC Data & Resource Center. https://doi.org/10.26275/sydt-lkiw

Pelot, N.A., Ezzell, J.A., Goldhagen, G.B., Musselman, E.D., Cariello, J.E., Clissold, K.A., Grill, W.M., 2020d. Pig Vagus Nerve Stained with Masson's Trichrome. Version 1, SPARC Data & Resource Center. https://doi.org/10.26275/pgr9-bk2e

Pelot, N.A., Ezzell, J.A., Goldhagen, G.B., Musselman, E.D., Cariello, J.E., Clissold, K.A., Grill, W.M., 2020e. Rat Vagus Nerve Stained with Masson's Trichrome. Version 5, SPARC Data & Resource Center. https://doi.org/10.26275/z3ab-7j9y
